# Understanding Basic Concepts of Viral Quasispecies: From Evolutionary Dynamics to Clinical Relevance

**DOI:** 10.3390/pathogens15080824

**Published:** 2026-08-05

**Authors:** Francisco Rodríguez-Frías, José Raúl Oubiña, David Tabernero, Maria Francesca Cortese, Josep Gregori, Maria Buti, Ariadna Rando-Segura, Josep Quer

**Affiliations:** 1 Department of Basic Sciences, Universitat Internacional de Catalunya, 08017 Barcelona, Spain; 2 Centro de Investigación Biomédica en Red de Enfermedades Hepáticas y Digestivas (CIBERehd), Instituto de Salud Carlos III, 28029 Madrid, Spain; maria.cortese@vhir.org (M.F.C.); mariabutiferret@gmail.com (M.B.); ariadna.rando@vallhebron.cat (A.R.-S.); josep.quer@vhir.org (J.Q.); 3 Instituto de Investigaciones en Microbiología y Parasitología Médica (IMPaM), Universidad de Buenos Aires-Consejo Nacional de Investigaciones Científicas y Técnicas (UBA-CONICET), Ciudad Autónoma de Buenos Aires 1121, Argentina; joserauloubina2012@gmail.com; 4 Universidad Católica de las Misiones (UCAMI), Posadas 3300, Misiones, Argentina; 5 Liver Diseases-Viral Hepatitis, Liver Unit, Vall d’Hebron Institut de Recerca (VHIR), Instituto de Investigación Sanitaria Hospital Universitari Vall d’Hebron (IIS IR-HUVH), Vall d’Hebron Barcelona Hospital Campus, 08035 Barcelona, Spain; josep.gregori@gmail.com; 6 Liver Unit, Microbiology Department, VHIR, IIS IR-HUVH, Vall d’Hebron Barcelona Hospital Campus, 08035 Barcelona, Spain; 7 Liver Unit, Internal Medicine Department, VHIR, IIS IR-HUVH, Vall d’Hebron Barcelona Hospital Campus, 08035 Barcelona, Spain; 8 Medicine Department, Universitat Autònoma de Barcelona (UAB), 08193 Bellaterra, Spain; 9 Virology Section, Microbiology Department, VHIR, IIS IR-HUVH, Vall d’Hebron Barcelona Hospital Campus, 08035 Barcelona, Spain; 10 Biochemistry and Molecular Biology Department, Universitat Autònoma de Barcelona (UAB), 08193 Bellaterra, Spain

**Keywords:** viral quasispecies, consensus sequence, master sequence, next-generation sequencing, diversity indices, antiviral treatment, resistance-associated substitutions, hepatitis B virus, hepatitis C virus, transcomplementation

## Abstract

This review examines the viral quasispecies concept and its implications for understanding viral evolution, pathogenesis, and the development of effective antiviral therapies. Quasispecies are dynamic populations of closely related but genetically distinct viral genomes, which evolve under mutation and Darwinian selection. Notably, minority variants, often dismissed as “genetic noise”, may harbor significant biological differences—such as drug resistance—and become dominant under changing selective pressures. Next-generation sequencing (NGS) has become an indispensable tool for characterizing genetic diversity within quasispecies, enabling detection and quantitative analysis of minority variants often missed by conventional Sanger sequencing, as well as the calculation of diversity indices. We highlight two NGS-based studies of hepatitis B virus quasispecies as illustrative examples of the relevance of minority variants in antiviral resistance and clinical outcomes. These studies also exemplify transcomplementation, whereby defective viral genomes can be replicated and packaged through functional proteins provided by co-infecting variants. Finally, we discuss how the quasispecies concept may extend beyond viruses, with parallels in biological systems such as the adaptive immune system and tumor cell populations. Recognizing quasispecies as dynamic evolving populations rather than static entities is crucial for developing successful strategies to address infectious diseases and other complex biological challenges.

## 1. Preliminary Observations

Let us consider the following hypothetical dialogue between a physician and a patient: “*We have initiated treatment for your chronic hepatitis B. The initial follow-up assessments have been very encouraging, with a substantial reduction in hepatitis B viral load, approaching undetectable levels. However, during the last control, we have observed a significant rebound in viral load. This suggests that the virus has mutated and is no longer sensitive to the current treatment. Nevertheless, we can redirect your treatment*”. While this scenario has become infrequent due to the great effectiveness of current antivirals against the hepatitis B virus (HBV), this dialogue contains two subtle but conceptually important inaccuracies that require clarification.

The first is the reference to “the virus” as an individual entity. This anthropomorphized simplification treats a heterogeneous viral population as a single entity, overlooking its biological and evolutionary dynamics. A more accurate representation should consider the virus not as a single agent, but as a genetically diverse and rapid evolving population within the host, named *quasispecies* [1]. An analogy found in natural world is the destructive power of army ants—known as *marabunta*—which is not the result of an individual ant, but rather the emergent behavior of a highly coordinated action of these eusocial insects, as well as the antiviral treatment resistance of viruses arises from the collective dynamics of viral populations [2]. This concept aligns with the idea of collective cognition in social insects [3], wherein the adaptability of the group exceeds the capabilities of any individual member, based on three fundamental events: a cooperative brood care, the overlapping of multiple generations with older offsprings helping to raise younger siblings, and reproductive division of labor. Understanding viral infections through the lens of population dynamics and evolutionary biology is essential for accurately interpreting the viral response to therapeutics and guiding effective combinations of antivirals for future strategies.

The second is the commonly—but potentially misleading—statement: “*the virus has mutated*”, which appears to refer to an event occurring during the course of treatment and caused by treatment. Nucleotide substitutions arise randomly in any replicating biological DNA or RNA system as a consequence of the errors introduced by the polymerases during the replication process. Since most RNA viruses replicate via RNA-dependent RNA polymerases, which do not have proofreading activity, they are unable to correct mistakes during replication. Consequently, RNA viruses generally exhibit higher mutation rates than DNA viruses [4]. Of note, mutation accumulation may be partially limited in some RNA viruses such as some members of *Nidovirales* order, including coronaviruses, which have an RNA polymerase independent proofreading activity [5]. On the other hand, mutation accumulation in some DNA viruses is comparable to that of RNA viruses which are replicated by RNA-dependent RNA polymerases lacking proofreading activity [6,7]. Mutations are continuously generated, especially in systems replicating at high levels as observed throughout the course of chronic HBV infection [8]. At the time of treatment, HBV genomes carrying antiviral resistance mutations can outcompete the drug-sensitive genomes, thereby becoming enriched in the quasispecies [9]. The result is that at treatment failure, changes are observed in genomic viral sequences.

In this review, we will explore the implications of the quasispecies nature of viral genomes. To begin with, it is worth recalling a statement by Charles Darwin. Though he was obviously unaware of the existence of viruses, he nevertheless remarked: “It seems pretty clear that organic beings must be exposed during several generations to the new conditions of life to cause any appreciable amount of variation [10]”. The genetic variation lies at the heart of the issue presented in the physician–patient dialogue. In fact, when the physician states that “*the virus has mutated*” what is essentially being described is that the viral population has undergone genetic variation. As Darwin observed in the context of natural selection—though he was unaware at the time of the molecular basis of heredity—organic beings possess heritable material. We now understand this material to be encoded in their genomes. The continuity of life across generations in living organisms is made possible through the replication of these genomes.

Viral replication relies on sophisticated mechanisms that ensure the faithful transmission of genetic information, thereby maintaining species identity. For example, hepatitis C virus (HCV) consistently produces progeny that are themselves hepatitis C viruses, a seemingly obvious but biologically remarkable feature that reflects the high precision of its replication process. However, if viral genomes are replicated with such fidelity, how do viruses manage to adapt to constantly changing environments? The answer lies in the occurrence of mutations, a concept that often evokes fear, partly due to movies and comic books. In scientific terms, mutations are simply changes in the genome (DNA or RNA), which can arise from replication errors, exposure to mutagens, or other factors. Despite their reputation, mutations constitute the raw material that enables both evolution and the adaptability of viruses to new conditions [11]. In fact, the evolution of all living organisms relies on mutations, which make adaptation to a changing world possible. In their absence, biological systems would lack the variability necessary to persist, and life on Earth might have amounted to nothing more than a short and unremarkable episode.

Therefore, in a very real sense, we owe our existence to mutations—we are, after all, the product of countless genomic alterations; “we are mutants!” Perfection, fortunately, does not exist, and genomic replication is no exception: It is imperfection that enables evolution [12]. Returning to our fictional dialogue between the physician and the patient, the final comment offers a hopeful note: “*We can redirect your treatment.*” This is not merely fiction; in clinical practice, such adaptability is often possible [13]. And it is made possible thanks to the accumulated knowledge about viruses and their genomic behavior, including their variability. This knowledge continues to guide therapeutic strategies and shape the future of precision medicine. Throughout this review, HBV will serve as the principal model system for illustrating concepts of viral quasispecies biology, complemented by selected examples from other viral systems.

### Simple Approach to Quasispecies Concept, Including Some Related Terms

The term quasispecies was coined in the 1970s by Manfred Eigen and Peter Schuster to refer to mutant distributions in populations of primitive replicons—i.e., any replicating entity—following a natural self-organization model named Hypercycles, enabling them to experience Darwinian evolution [14,15]. Soon after, the existence of mutant distributions similar to those of primitive replicons was experimentally observed in bacteriophage Qβ populations [16], providing evidence of quasispecies organization in RNA viruses. In such viruses, a large fraction of progeny sequences is expected to contain one or more mutations relative to their parental templates in each replication cycle, ultimately resulting in a highly heterogeneous population. Therefore, a viral quasispecies can be considered as a swarm—also termed “spectra” or “cloud”—of variants within a host, among which those with a biological advantage during replication are selected, following a strict Darwinian process [1]. While Darwinian evolution occurs in both species—i.e., cellular organisms—and quasispecies, the latter display it at a strikingly rapid pace. As a result, within a single infected individual, changes in the composition of the viral population can be observed over short timescales, and, unfortunately, their direct consequences become evident when environmental conditions impose new selective pressures—for instance, the implementation of an antiviral treatment. For this reason, the study of quasispecies dynamics is important to understand the adaptability, pathogenicity, and persistence of viruses, and to design strategies to prevent and treat the diseases they cause.

This remarkable adaptability of quasispecies contrasts sharply with the limited capacity of adaptation in species. From an evolutionary perspective, a species is constituted by a unique sequence since parents and offspring share nearly identical genomes, due to the low error rate of the replication mechanisms—for instance, in human genes the rate of spontaneously arising mutations is 10^−9^ substitutions per nucleotide and generation [17]. This strongly contrasts with quasispecies, which cannot be described by a single genome sequence, but rather using concepts such as *average sequence*—also known as *consensus sequence*—or *majority or predominant sequence*—best known as *master sequence*. In relation to these “strange concepts”, they are defined as stated below [2]:

- *Average or consensus sequences:* They are obtained by selecting, at each position of an aligned set of homologous sequences, the nucleotide—or amino acid—that appears most frequently. The consensus sequence does not necessarily correspond to any individual genome in the mutant spectrum. Moreover, the consensus sequence may not capture all biologically relevant genetic variation present within a quasispecies. For instance, in a recent study some of the most frequent mutations identified in SARS-CoV-2 quasispecies were never represented in published consensus genomes, likely because they are predicted to be incompatible with viral viability [18].
- *The master or majority sequence:* This refers to the most abundant genomic sequence in a quasispecies. It generally shows a selective advantage of this sequence relative to the other components of the quasispecies. It may coincide—or not—with the consensus sequence.

Therefore, these two concepts may not coincide with each other, a quasispecies can have an average sequence very different from the majority sequence. In fact, the master sequence may even change over time due to the selection of a variant better suited to the new environment, in the classic Darwinian process of survival of the fittest.

In the literature on quasispecies, it is usual to find additional concepts and terms related to this situation [2]. In this sense, the concept of *fitness* refers to a parameter that quantifies how well an organism or a virus adapts to a specific environment. It is necessarily a relative measure. In the context of viruses, *relative fitness* measures their capacity to produce infectious progeny relative to a reference viral clone, in a defined environment. A related notion, *epidemiological fitness* describes the relative ability of a virus to outcompete other circulating variants and become dominant in the field during, or as a consequence of, an epidemic. Other key concepts related to variant selection are the *mutant spectrum* which encompasses the entire collection of mutant genomes within a quasispecies, which must be characterized by quantifying the proportions of its different *haplotypes*—sets of identical sequences. In this regard, *mutation frequency* denotes the proportion of mutant genomes within the overall population. It can be computed for an entire sequence or for a specific site of a genome.

It is tempting to dismiss the viral genomes of a quasispecies that are in very small proportions, considering them mere “genetic noise” resulting from high mutation rates. These supposedly irrelevant proportions are assumed arbitrarily, since there is no objective data available to indicate which proportions may be relevant. However, reality has shown that such minority genomes can even exhibit very dissimilar biological behavior from that shown by the main components of the populations in which they are observed: virulent versus attenuated, antiviral-resistant variants associated with treatment failures versus antiviral-sensitive variants, or antibody-resistant variants associated with vaccine or immunotherapy failures versus antibody-sensitive variants, etc. [19]. This hidden potential for phenotypic variation becomes relevant in the context of positive or negative selection of a variant as well as the random generation of new mutations due to the high error rate of their polymerases and replicative systems—usually assumed to be due to lack of replication error correction capacity.

Indeed, these omnipresent evolutionary forces can shift the balance within a viral population, allowing minority genomic variants to become dominant and relegating previously dominant forms to the minority. The viral population, conceptualized as a quasispecies, can undergo rapid evolutionary changes, potentially occurring over the span of hours or even minutes. Nonetheless, the role of the temporal dimension in shaping quasispecies dynamics remains poorly understood, and disregarding minority variants may be equivalent to “sweeping them under the rug” [20]. Faced with this reality, we must pursue the most precise knowledge possible of the composition and organization of viral quasispecies, together with the quantification and biological significance of their intrahost genetic diversity. The quasispecies concept applies to all RNA viruses studied to date, as well as to DNA viruses whose replication is affected by low-fidelity viral polymerases—e.g., HBV reverse transcriptase [6] and parvovirus B19 nonstructural protein [7]—by error-prone viral DNA repair pathway—e.g., African swine fever virus [21]—and additional cellular mechanisms—e.g., APOBEC family proteins, and other mutagenic factors [21,22,23,24,25].

## 2. How Can We Characterize a Quasispecies?

Here, we discuss basic concepts and some of the most useful diversity and complexity calculations—indices. The most academically strict approach to characterize a population of genomes is to sequence all its individual components. This is analogous to analyzing the biodiversity of a forest by identifying and mapping every organism within it. While such exhaustive analysis is practically unfeasible, ecologists have developed strategies to overcome this limitation—for instance—conducting sectorial analysis through marked transects and using computational tools to extrapolate data to the entire ecosystem [26,27]. Similarly, when studying the diversity of viral genomes in an infected patient, it is not possible to isolate and sequence the entire viral population of the liver, for obvious technical and biological reasons. However, we can obtain representative samples, such as a few milliliters of plasma or tissue biopsies, and apply deep-sequencing methodologies and computational tools from ecology. There is no need to “reinvent the wheel”; instead, we can build on the well-established expertise of other scientific disciplines.

**Figure 1:**
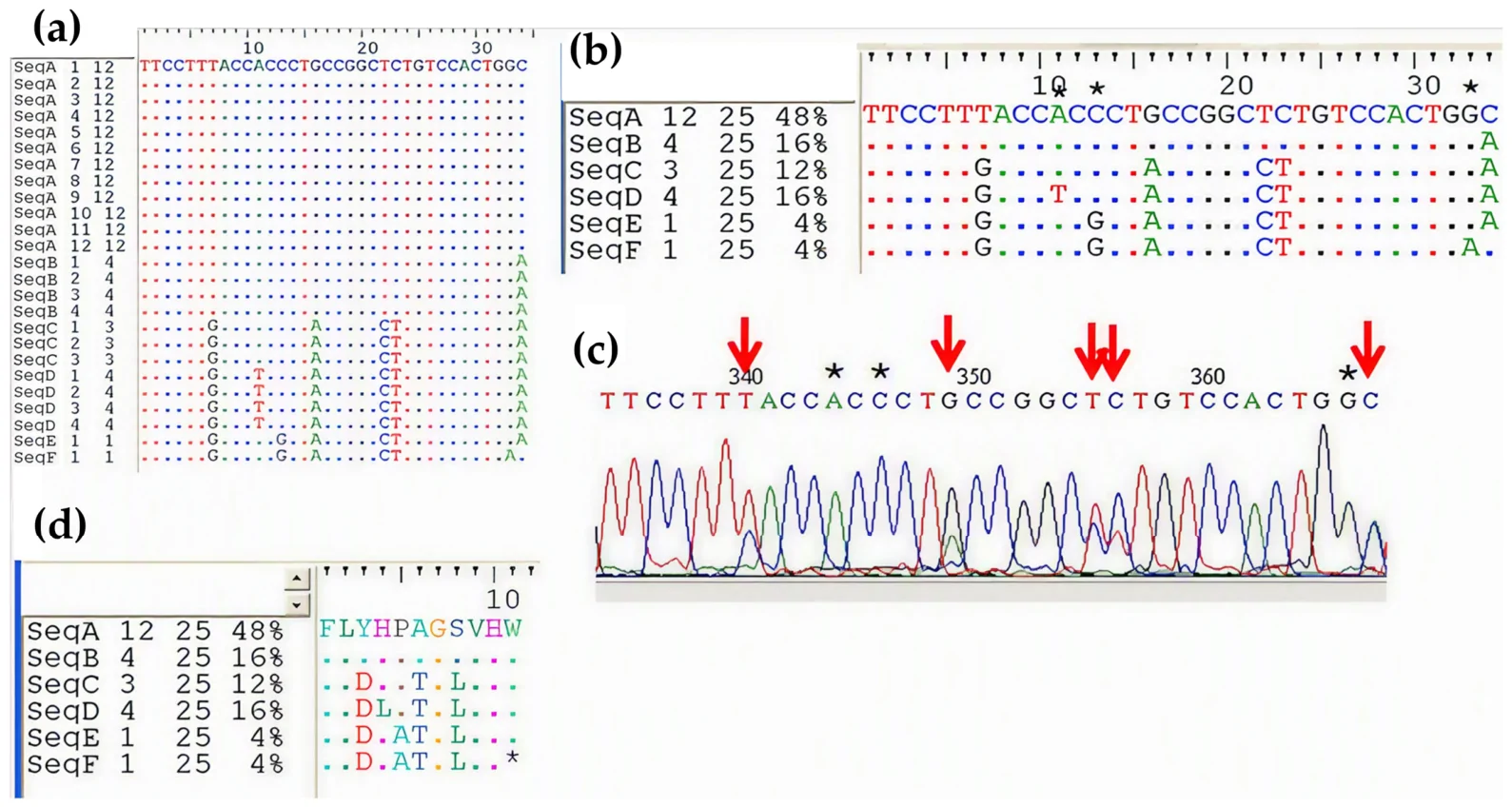
Example of a typical quasispecies profile. (**a**) Sequence alignment of 25 clones from the region between nucleotide positions 2031 to 2064 of the HCV genome. (**b**) Collapsed clone sequences were grouped into haplotypes—a single sequence representing each group of identical sequences. For each haplotype, the number of identical sequences, the total number of clones sequenced, and the proportion of each haplotype are indicated. (**c**) Population sequencing—Sanger—electropherogram of the sample that was processed using clones. Positions with more than one nucleotide are indicated with red arrows, signifying the presence of sequences—variants—within the quasispecies at proportions detectable by Sanger sequencing. Note that some of the clone sequences—sequences E and F—are not detected by Sanger sequencing, as their proportion is lower than the detection limit of this method. (**d**) Translation into amino acids of the haplotypes depicted in panel b, note that one of the clones—Seq F—shows a newly created stop codon. (*) Identifies the same positions in panels (**b**,**c**). However, in panel (**d**), it indicates "Stop," as shown in the panel itself.

Figure 1 provides an illustrative example of the heterogeneity and complexity of a viral quasispecies. This example shows an alignment of 25 sequences obtained using molecular cloning, from a region of the HCV genome (Figure 1a), revealing six distinct genomic clusters. The largest cluster consists of 12 identical sequences without any mutations and is designated Seq A, which represents the master sequence. Other clusters include, for example, four sequences carrying the C34A substitution—Seq B—among others. Each group of identical sequences is collapsed into a single representative haplotype. Figure 1b displays all nucleotide haplotypes identified within the sequenced sample, each comprising a number of identical genomes. Their relative abundance within the total population defines their frequency. Figure 1d displays the translation of these nucleotide haplotypes into their corresponding amino acid sequences. By contrast, if Sanger sequencing is performed at population level—without a previous cloning step—it provides only a qualitative view of the intrahost genetic diversity underlying the quasispecies complexity. The electropherogram obtained by Sanger sequencing of the original sample from which the clones were obtained (Figure 1c) yields a single sequence representing the consensus of the viral population. Mixed nucleotide peaks may be observed; however, they do not allow determination of the relative proportions of each variant, nor whether the observed substitutions occur in the same haplotype. These limitations are consistent with observations from other conventional methods, including restriction fragment length polymorphism (RFLP)-based genotyping assays [28]. In fact, Sanger sequencing, RFLP, and other conventional methods are low-throughput methods that lack the sensitivity to reliably detect most minority mutations identified by molecular cloning (see asterisks in Figure 1b,c). As such, many variants present at low frequencies in the population remain undetected by these approaches.

**Figure 2:**
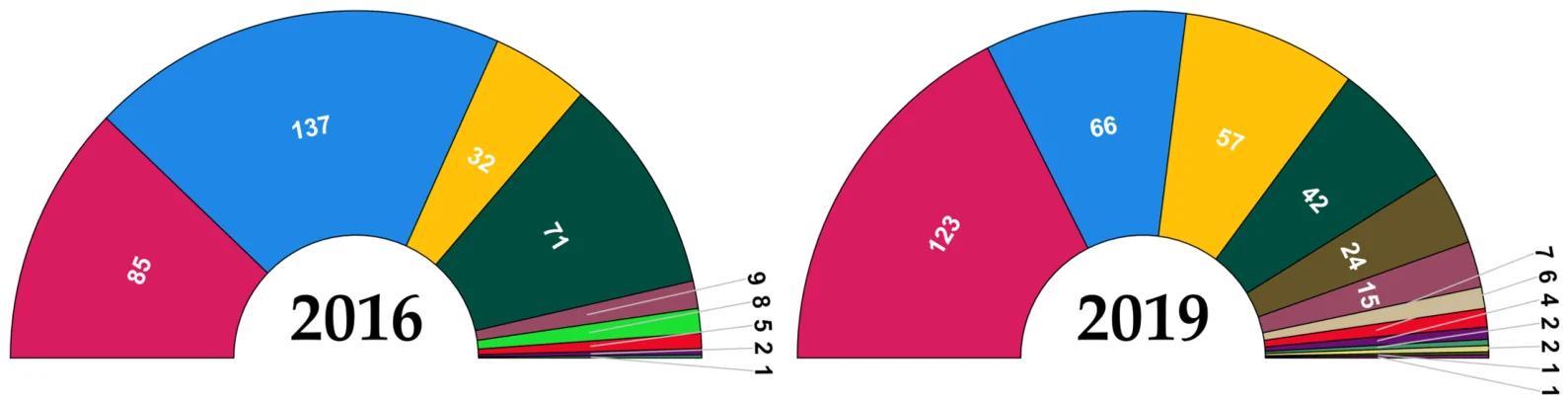
Distribution of seats in the Spanish parliament after the general elections of 2016 and 2019. Data are based on publicly available electoral results [31]. This non-biological example is used as an illustrative analogy for the calculation of Shannon’s entropy, a widely used diversity index for quantifying genetic diversity within viral quasispecies, by analogy with populations composed of discrete groups with defined relative abundances.

As commented above, we can study the diversity of the viral genomes within an infected patient by obtaining representative biological samples and applying deep-sequencing methodologies together with computational tools originally developed for ecological studies. To investigate genomic variation, the most powerful tool currently available is high-throughput sequencing, commonly referred to as next-generation sequencing (NGS). This technology enables the generation of thousands to millions of sequences, known as reads, from a given viral population, providing an unprecedented resolution of its genetic makeup. The resulting data can be rigorously analyzed using a multidimensional framework, based on various and complementary diversity indices and quasispecies structure indicators to characterize intrahost genetic diversity and quasispecies organization [29,30]. One of the best-known diversity indices is Shannon’s Entropy (H), which takes into account the relative proportion of each haplotype or group [30]—like those indicated Figure 1b. Because Shannon’s entropy is a purely mathematical measure of diversity, this calculation can be applied to any population composed of discrete groups with known relative abundances, regardless of their biological or non-biological nature [31]. To illustrate how this index reflects the diversity and relative distribution of population components, we use a simple non-biological example: the parliamentary seat distributions among political parties after general elections. Accordingly, H was calculated using publicly available data from the 2016 and 2019 Spanish general elections [31] (Figure 2), treating each political party as a discrete category (analogous to a viral haplotype) and its number of seats as a proxy for relative abundance. Entropy was higher in 2019 (H = 0.73886) than in 2016 (H = 0.65105), indicating a reduction in the dominance of any single party and a more even distribution of representation across groups. As a consequence, the collective behavior of the system becomes less predictable and more dependent on cooperative interactions, which may cause greater difficulty in managing the legislative process of the parliament. In this illustrative analogy, the 2019 parliament represents a system that is more heterogeneous and less predictable than that of 2016, closely mirroring the behavior of a viral quasispecies with higher entropy.

Exchanging the results—that is, number of deputies—between the two most represented parties depicted in Figure 2, while maintaining the same numerical proportions, would not change the H index, but the resulting parliament would show a different functional behavior. This example demonstrates that the H index is not sufficient to fully describe a complex population, such as a quasispecies. For this reason, additional indices have been defined, including mutation frequency (Mf) or Nucleotide Diversity (Q), that allow the consideration of the differences between the components of the quasispecies, rather than their relative composition. While Mf is defined as the fraction of nucleotides in the population that differ from the dominant haplotype (the master sequence), Q takes into account the pairwise differences between haplotypes and is strictly defined as: average fraction of nucleotide differences between pairs of sequences in the population [30]. Another intuitive diversity measure is the Gini–Simpson index, which quantifies inequality among haplotypes within the same sample. It can be interpreted as the probability that two viral genomes randomly drawn from a given quasispecies belong to different haplotypes; values closer to 1 indicate greater structural diversity and, therefore, a more complex quasispecies structure [30]. However, these classical indices may fail to fully capture the relationship between genetic diversity and phenotypic outcomes, such as the average fitness of a quasispecies or its capacity to evade antiviral treatments. In this context, we have recently reported observations of unprecedented genetic diversity coexisting with preserved functionality in fast-evolving hepatitis E virus quasispecies when treated with the mutagen ribavirin [32]. This underscores the need for innovative evolutionary metrics—such as *quasispecies maturity*, *haplotype synonymy*, *genetic amplitude*, and *flat-like quasispecies*—to better describe the interplay between genetic diversity and phenotypic functionality of rapidly evolving systems [33]. Recent work [34] further suggests that quasispecies evolution may follow a general transition from peaked towards flat-like quasispecies structures. While peaked structures are usual in short-lived viral infections, which display low genetic diversity and strong dominance by one or few haplotypes, flat-like structures are characteristic of long-lasting chronic infections, with high genetic diversity, no clearly dominant haplotypes, and high evenness. Mature quasispecies are closer to this latter state, which results in increased fitness and resilience. In the same work, a normalized quasispecies maturity score was proposed to place a quasispecies along this evolutionary structural trajectory. At the core of this approach lies a fundamental principle: heterogeneity and complexity are intrinsic to viral infections. Understanding these infections—and their implications for human health—is not possible without acknowledging this reality.

Although these diversity and evolutionary metrics provide valuable insights into quasispecies dynamics and evolutionary trajectories, their application in routine clinical practice remains limited. Broader clinical implementation of NGS-derived evolutionary metrics will require standardized laboratory protocols, harmonized analytical workflows and clinically validated interpretation criteria. Consequently, prospective studies performed under these standardized conditions will be necessary to validate the relationship between evolutionary metrics, such as quasispecies maturity, and clinically meaningful outcomes.

Recent advances in sequencing technologies continue to expand the analytical capabilities for characterizing viral quasispecies and their intrahost genetic diversity. Beyond conventional short-read NGS, third-generation sequencing (TGS) technologies generate substantially longer reads, enabling improved characterization of genetically complex viral populations and facilitating the reconstruction of full-length viral haplotypes [35]. These approaches have already been successfully applied to viruses including HBV and SARS-CoV-2 [36,37]. Of note, accurate reconstruction of individual, full-length viral haplotypes from TGS data is hampered by its high sequencing error rate relative to second-generation short-read platforms [38]. For this reason, novel computational methods focused on generating haplotype-resolved viral genomic sequences from TGS data have been developed [39]. Furthermore, emerging NGS technologies, such as single-cell sequencing approaches [40], are beginning to expand the study of viral quasispecies.

Nevertheless, NGS-based characterization of viral quasispecies remain influenced by methodological factors affecting both sample preparation and data analysis. Among factors related to sample preparation PCR amplification is particularly important, which may bias the relative frequencies of variants due to differences in primer binding affinities and resampling of individual variants. In addition, chimera formation and polymerase errors during PCR create new artifactual variants. Regarding sequencing errors, appropriate statistical and bioinformatic error correction is crucial to accurately infer the genetic diversity within quasispecies [41,42]. Consequently, careful experimental design together with appropriate bioinformatic processing is essential for obtaining reliable measurements of viral quasispecies composition and genetic diversity and defining robust thresholds for low-frequency variant detection and interpretation.

## 3. The Quasispecies Concept: Beyond Viral Evasion

According to the *Oxford Dictionary of Biochemistry and Molecular Biology*, a quasispecies is defined as a “population of closely related but different viral genomes that evolves over time through spontaneous mutations within an individual infected with a single genotype. Diversification through the emergence of quasispecies is one mechanism by which viruses evade the host immune response [43]”. This definition, however, does not explicitly mention that, beyond the evasion of the host immune response, viral evolution can be driven by a wide range of additional factors. These include antiviral treatment, biochemical shifts, or virtually any environmental change. In fact, viral evolution occurs even in a seemingly stable cell culture, without the presence of overt external selective pressures, mainly by two fundamental properties of RNA and certain DNA viral populations: the continuous generation of new mutations, produced by the low replicating fidelity of the replication machinery in these viruses, and the ongoing selection of variants driven by quasispecies internal fitness gradients [14], together with viral competition [44]. The continuous quasispecies diversification even in the absence of external selective forces has been documented in experimental data from HCV replication systems. This diversification was driven by internal disequilibria and haplotype fitness gradients, and enhanced genetic diversity, rather than convergence towards a new consensus sequence, improved quasispecies fitness, and promoted resistance to direct-acting antivirals (DAAs) and mutagens [45,46,47,48,49]. Therefore, viral evolution can only be effectively halted under conditions where replication is suppressed, such as outside the host cell or under extreme physical constraints—e.g., freezing the virus.

**Figure 3:**
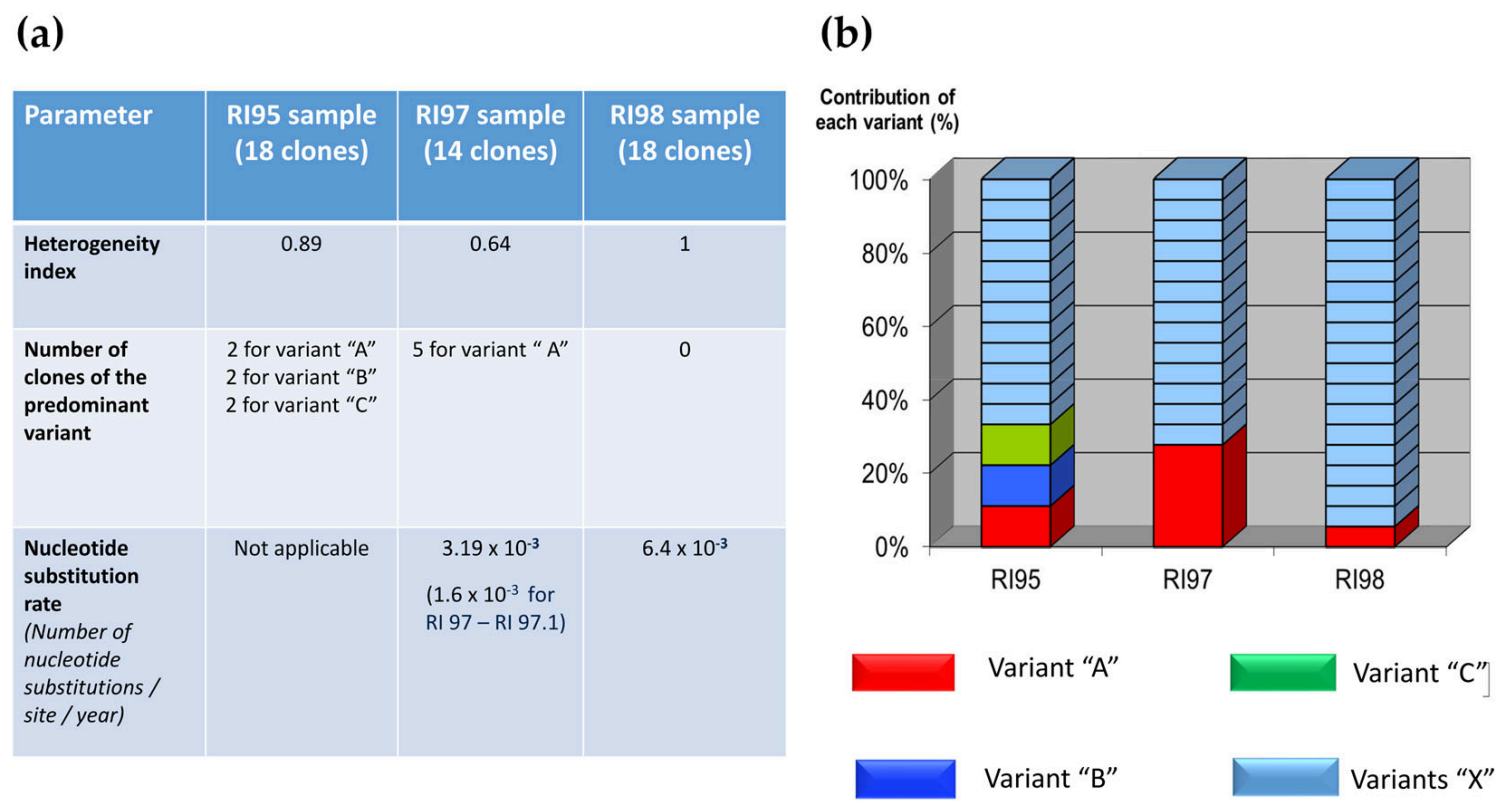
Dynamics of HBV quasispecies in a chronically infected patient (RI). HBV quasispecies dynamics were analyzed by gene cloning in a chronically infected patient, who was followed-up throughout a three-year observation period—1995–1998—without receiving antiviral treatment. Quasispecies populations are labeled according to the year of sampling—e.g., RI95 means the quasispecies population observed in 1995. (**a**) Genetic heterogeneity of HBV variants based on the analysis of fifty clones, obtained by PCR amplification of the S gene from circulating genomes in patient’s plasma and subsequent amplicon cloning. Two clones in 1995, five in 1997 and one in 1998 shared identical sequences. In 1998 all clones exhibited different sequences, and therefore no predominant—i.e., master—sequence could be demonstrated at this time point. RI97.1 designates the variant 1 obtained in the sample RI97, commented in Figure 4. (**b**) Contribution of variants throughout the observation period. Variant “A”—in red—corresponded to the ancestor sequence (see Figure 4), which was detected at all time points studied (RI95, RI97 and RI98). Variant “B”—in blue—and “C”—in green—were only detected in the first sample (RI95). Variants labeled “X”—all shown in sky blue—represent distinct low-frequency variants, each detected as a single sequence. Data obtained from Virus Research, 123, Mathet V.L. et al., Dynamics of a hepatitis B virus e antigen minus population ascribed to genotype F during the course of a chronic infection despite the presence of anti-HBs antibodies, Pages No. 72–85, Copyright 2007, with permission from Elsevier [50].

This principle of quasispecies dynamics and their adaptive potential is particularly relevant in chronic viral infections, where viruses must adapt to host immune responses and antiviral pressures over extended periods. A clear example of such intrahost adaptive evolution was documented several years ago [50]. Notably, this study reported, for the first time, viral evolution in an HBV genotype F chronically infected patient (RI), despite the simultaneous detection of circulating hepatitis B surface antigen (HBsAg) and anti-HBs antibodies (Figure 3). The patient, who suffered from chronic active hepatitis and cirrhosis, was followed up over a three-year period (1995–1998).

**Figure 4:**
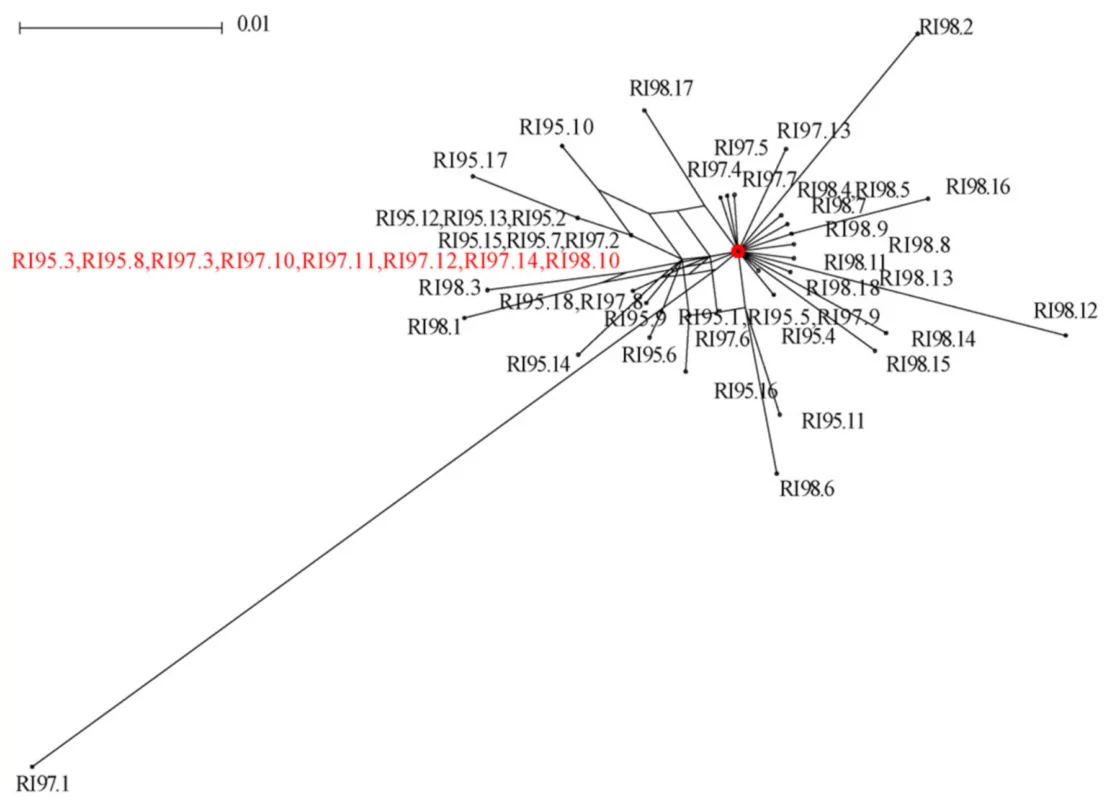
Split decomposition of all 50 clone-derived sequences obtained from RI95, RI97, and RI98 samples. Each variant was named using a code that denotes the sample and the variant number—e.g., RI95.1. Red-colored codes show identical ancestral sequences—represented as Variant “A” in Figure 3—whose location in the split network is indicated by a red node. Bar lengths represent the specified genetic distances. Higher genetic distances were observed among several sequences from RI98 compared with those from RI95 and RI97. Nevertheless, the most divergent sequence corresponded to a single clone from RI97 (RI97.1), where 17 mutations were recorded, whereas the remaining 13 clones derived from RI97 exhibited no more than two nucleotide changes per clone. The aforementioned unusual sequence also contained a stop codon at the 3’ end of the S gene—amino acid position 156—together with additional mutations not detected among the remaining clones, suggesting a defective variant within the quasispecies. See Section 3.3 for further discussion on the presence of defective variants in the quasispecies. Reprinted from Virus Research, 123, Mathet V.L. et al., Dynamics of a hepatitis B virus e antigen minus population ascribed to genotype F during the course of a chronic infection despite the presence of anti-HBs antibodies, Pages No. 72–85, Copyright 2007, with permission from Elsevier [50].

**Figure 5:**
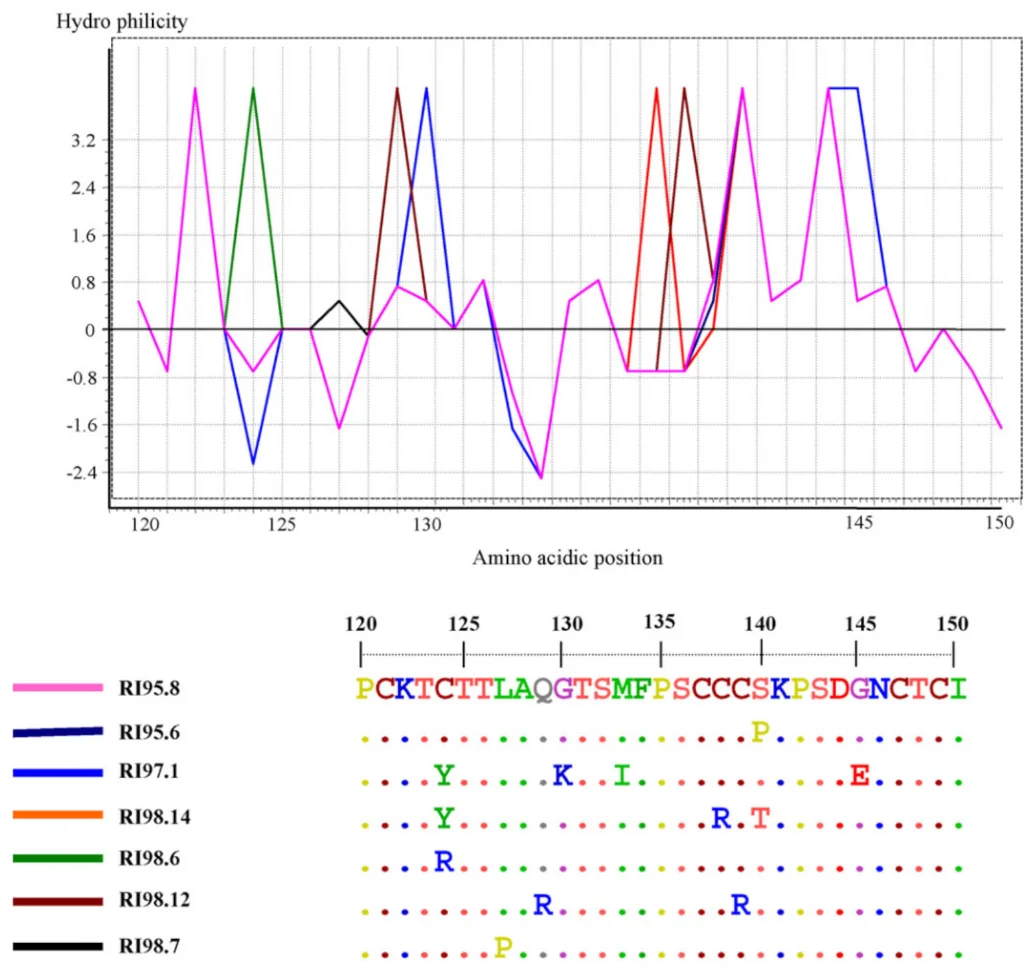
Hydrophilicity patterns obtained for HBV RI clones, named according to the sample and the variant number—e.g., RI98.14—with non-synonymous mutations relative to the ancestral sequence (RI95.8). A partial analysis of the S protein—amino acid positions 120–150—encompassing the “*a*” determinant, is shown. Some of these substitutions are well-known changes able to modify the conformational structure of the “*a*” determinant—e.g., C124Y, C124R and G145E. Reprinted from Virus Research, 123, Mathet V.L. et al., Dynamics of a hepatitis B virus e antigen minus population ascribed to genotype F during the course of a chronic infection despite the presence of anti-HBs antibodies, Pages No. 72–85, Copyright 2007, with permission from Elsevier [50].

In such chronic scenarios, the continuous generation and selection of diverse viral variants within the quasispecies (Figure 4) enabled the virus to persist and evade immune surveillance. This was shown by both crucial amino acid changes within and outside the “*a*” antigenic determinant in the major hydrophilic region of the S protein (Figure 5) associated with the intermittent detection of typical neutralizing anti-HBs antibodies. In parallel, substitutions were also observed within Pol-derived CD8^+^-specific T cell epitopes.

**Figure 6:**
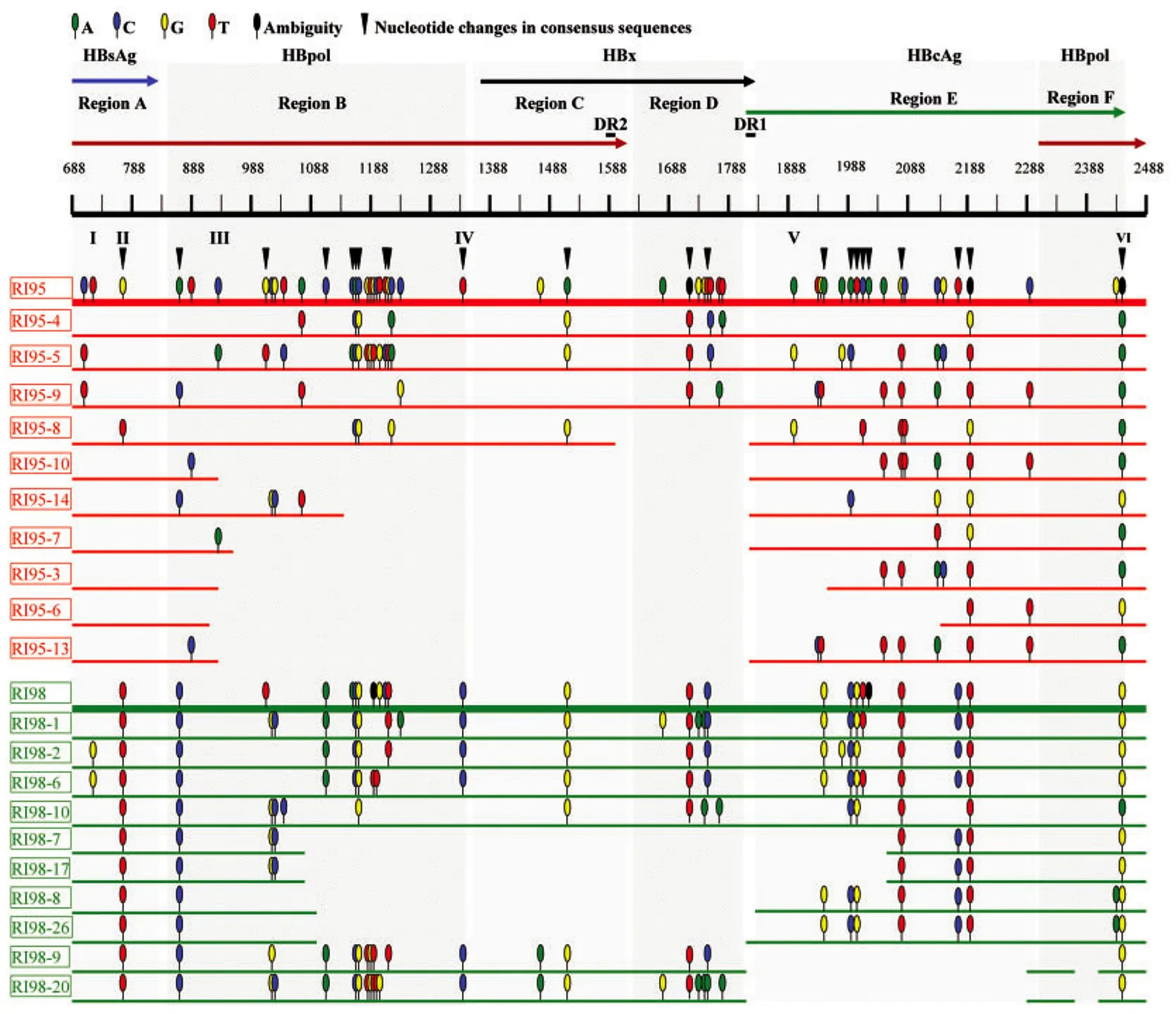
Analysis of the HBV population heterogeneity in plasma samples from patient RI obtained in 1995 (RI95) and 1998 (RI98). Cloning and sequencing of a genomic fragment spanning nearly half of the genome were performed, with each clone named according to the sample and the variant number—e.g., RI95-4. All analyzed sequences were assigned to genotype F, clade Ib. Intriguingly, a substantial proportion—approximately two-thirds—of the analyzed clones exhibited long nucleotide deletions, indicating the presence of defective genomes within the circulating quasispecies. See Section 3.3 for further discussion of defective variants in the quasispecies. Reprinted from Journal of General Virology, 88, Lopez J.L. et al., Intrahost evolution of HBe antigen-negative hepatitis B virus genomes ascribed to the F genotype: a longitudinal 3 year retrospective study, Pages No. 86–91, Copyright 2007, with permission from the Microbiology Society [51].

In another study [51], nearly full-length HBV genome sequences obtained in 1995 (RI95) and 1998 (RI98) from the above-mentioned patient RI revealed that 26 mutations accumulated over a three-year period—corresponding to a mutation of rate 2.7 × 10^−3^ substitutions per site per year. These changes were characterized by a predominance of non-synonymous substitutions, which were preferentially placed within single coding regions. In addition, 10 nucleotide ambiguities suggested the presence of mixed populations. To assess population heterogeneity, a genomic fragment spanning approximately half of the HBV genome was cloned and 20 clones derived from RI95 and RI98 were sequenced. Remarkably, nearly two-thirds of the analyzed clones contained large nucleotide deletions (Figure 6), highlighting the presence of defective genomes within the circulating population. Pairwise genetic distance analyses among these clone-derived sequences further indicated higher diversity among RI95 clones compared with those from RI98. Altogether, these findings support the existence of an HBV quasispecies displaying high intrahost genetic diversity in patient RI, a feature that may have contributed to long-term viral persistence.

### 3.1. The Quasispecies Concept: A Community of Viral Variants

As discussed in Section Simple Approach to Quasispecies Concept, Including Some Related Terms, a viral quasispecies should not be viewed as a single, monolithic entity but rather a complex cloud of closely related, yet distinct, genomes. This diversity arises from high mutation rates characteristic of many RNA viruses and certain DNA viruses, including hepatotropic viruses such as HBV and HCV. Within this population, individual genomes often differ by one or more nucleotides. When confronting such a dynamic *mutational cloud*, understanding its complex structure becomes paramount, as it can profoundly influence therapeutic success. Our struggle against viral infections can be likened to a strategic battle; as Sun Tzu articulated in *The Art of War*, “If you know the enemy and know yourself, you need not fear the result of a hundred battles [52]”. In the context of hepatotropic viruses, this “enemy” is the viral quasispecies itself—an intricate reservoir of variants with an extraordinary capacity to adapt to diverse pressures, including antiviral treatment. Critically, variants with reduced sensitivity to treatment may pre-exist within this population, even before the treatment is initiated—or even designed. These *resistant variants* can then be freely replicated and undergo Darwinian selection in the presence of the antiviral, leading to treatment failure [53,54].

To illustrate quasispecies dynamics, consider an anecdote from a summer night Mozart concert under a luminous moon, attended by 10,000 people. In a surprising turn of events, the concert was abruptly disrupted by an unexpected, artificially induced downpour. This sudden rain, orchestrated by a Mozart non-lover aiming to disperse the crowd, forced most of the attendees to leave. However, a small, unaware but resilient subset—approximately 0.1% of the audience—remained unfazed. By sheer chance, these 10 individuals had come prepared with umbrellas or hoods, allowing them to weather the unexpected storm and continue their enjoyment of the disrupted concert. These individuals, by chance, exhibited traits that conferred resistance to the environmental pressure imposed. Over time, the original attendees returned, now equipped with rain protection, rendering the initial disruptive strategy ineffective. This metaphor mirrors the quasispecies model, in which a population, although initially homogeneous in appearance—in the concert example, 99.9% homogeneous, without umbrellas nor hoods—still contains rare variants—0.1% random owners of umbrellas or hoods—that provide a selective advantage under stress. The concertgoers represent a “human quasispecies”: a dynamic population capable of rapid adaptation via selection of pre-existing, spontaneously occurring phenotypic variants. Such adaptability, driven by environmental pressure, leads to shifts in population composition and the emergence of resistance, providing an intuitive framework for understanding viral evolution, immune escape, and antiviral treatment resistance.

### 3.2. Escape from Drug Therapy

The situation described in the previous example is similar to maintaining antiviral treatment in HBV infection once resistant variants have been selected. In this setting, therapy is mainly based on the use of inhibitors of the viral polymerase, such as nucleos(t)ide analogues (NUCs) [55]. Among the most prominent and well-known variants non-sensitive to these inhibitors—i.e., resistant to them—are those carrying the substitution methionine to valine at position 204 (rtM204V) within the YM_204_DD motif—putative active site—located in the catalytic domain C of the HBV reverse transcriptase, which is thereby converted to YV_204_DD. This substitution confers resistance to NUCs such as lamivudine (LMV), a nucleoside analogue used in both human immunodeficiency virus (HIV) and HBV infections [56]. The selection of this variant is evidenced by the increase in blood levels of HBV DNA—viral load—after an initial decrease in viral load, a phenomenon known as *virological breakthrough*, which is typically followed by an elevation in liver enzymes levels—e.g., alanine amino transferase, ALT—[13]. Although genomes harboring the rtM204V mutation replicate less efficiently than the YMDD-containing polymerase variants—known as Wild Type, WT—[57,58], they can persist at low frequencies within the quasispecies as rare haplotypes in treatment-naïve patients. These minor populations will be detectable only by means of highly sensitive sequencing methods, such as NGS technologies [53,54]. These minor populations are selected when the LMV treatments are applied resulting in treatment failure—a phenomenon known as *antiviral resistance* [59]. Therefore, in the clinical discussion between our hypothetical physician and the patient, it is often imprecise to state that “*the virus has mutated*” as the cause of treatment failure, thereby misattributing the treatment failure—i.e., viral relapse—to a de novo viral mutation. A more accurate interpretation is that variants carrying mutations that allow the virus to escape antiviral pressure were already present within the viral quasispecies prior to treatment. These variants arise through the continuous generation of mutations during viral replication, favored by the high replication rate and lack of proofreading mechanisms during viral replication and are selectively amplified under therapeutic pressure.

Treatment failure is particularly frequent with antivirals that have a low genetic barrier to resistance, a concept that refers to the inherent difficulty with which a virus develops resistance to a specific antiviral, due to the number of RAS necessary to produce a significant decrease in susceptibility to this antiviral [59]. For instance, LMV has a low barrier against HBV antiviral resistance since the main variant responsible for resistance to this treatment involves a single substitution, rtM204V, which undergoes intense selective pressure during treatment, with 70% of patients experiencing antiviral resistance after 5 years of treatment [60]. Similarly, in the context of HCV infection, first-generation DAA targeting the NS3 protein also showed a low genetic barrier, and multiple variants with different levels of resistance have been described in vitro and in vivo for different HCV DAAs [61,62,63]. In fact, NS3 protease inhibitor RAS have been detected at varying frequencies as pre-existing variants in treatment-naïve patients using direct and cloning sequencing analyses [64,65,66,67,68], and subsequently with higher sensitivity through NGS [19].

**Figure 7:**
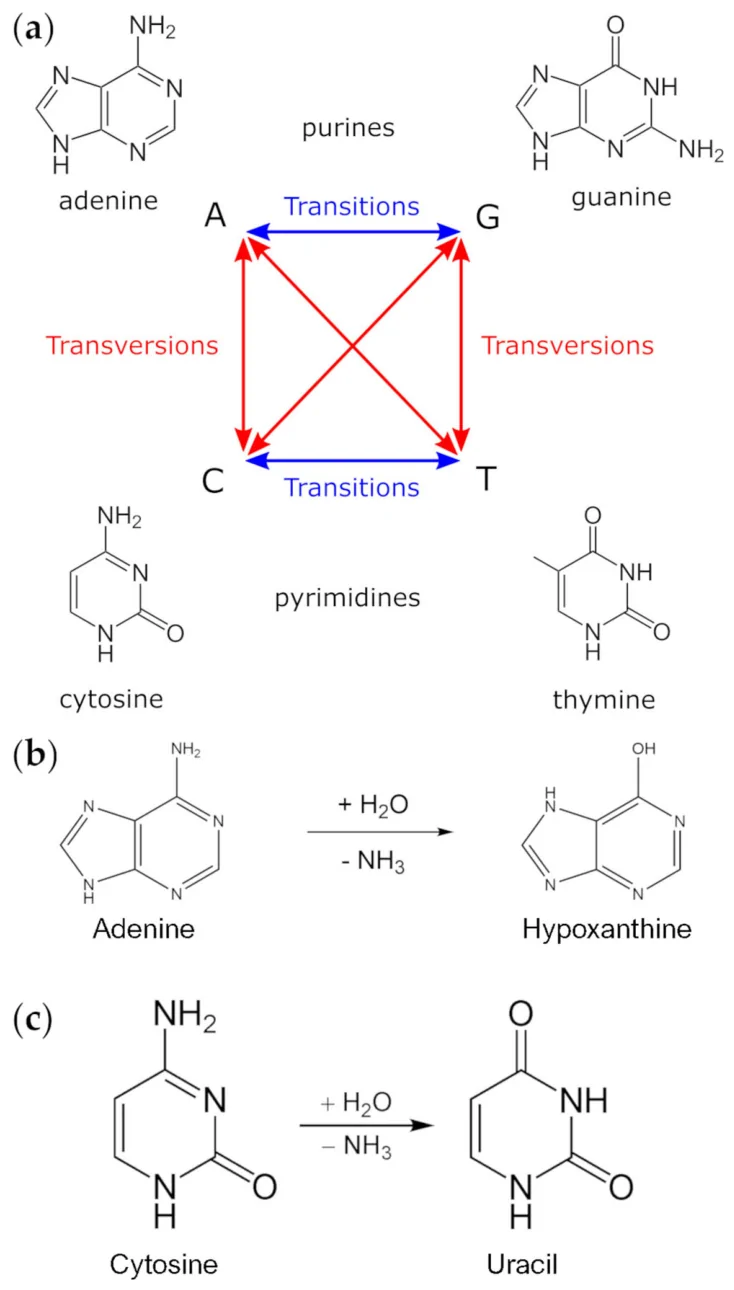
Mutation mechanisms: origin of transversions and transitions. (**a**) Schematic representation of all possible base substitutions. Transitions, in which a purine is replaced by another purine or a pyrimidine by another pyrimidine, are indicated with blue arrows. Transversions, in which a purine is substituted by a pyrimidine or vice versa, are indicated with red arrows. Image by Petulda-Own work, CC BY-SA 4.0, https://commons.wikimedia.org/w/index.php?curid=45586369 (accessed on 3 June 2025). (**b**) Deamination of adenine produces the purine derivative hypoxanthine, a constituent of the nucleoside inosine. Upon replication of viral genomes, this modification results in A → G transitions [25,73]. This process is mediated by the cellular family of RNA editing enzymes ADAR (adenosine deaminase acting on RNA), principally ADAR1. Image adapted from that by Grobert1234-Own work, CC BY-SA 4.0, https://commons.wikimedia.org/w/index.php?curid=63557232 (accessed on 3 June 2025). The names adenine and hypoxantine have been added beneath the corresponding bases. (**c**) Deamination of cytosine yields uracil, which pairs with adenine [25]. This alteration leads to C → T transitions after replication. This process is mediated by enzymes of the APOBEC (apolipoprotein B mRNA-editing enzyme catalytic polypeptide-like) family. Image adapted from that by Yikrazuul-Own work, Public Domain, https://commons.wikimedia.org/w/index.php?curid=3513581 (accessed on 3 June 2025). The names Cytosine and Uracil have been added beneath the corresponding bases.

Typically, RAS involves a minimal number of nucleotide changes that confer phenotypic resistance. The probability that a specific nucleotide change will arise within a given genome varies significantly according to the type of substitution. For instance, a purine-to-purine—e.g., A to G—or a pyrimidine-to-pyrimidine—e.g., C to T—replacement constitutes a *transition*, which generally arises with higher probability. In contrast, a purine-to-pyrimidine or a pyrimidine-to-purine replacement leads to a *transversion*, involving a more radical chemical change and occurring at much lower frequency (Figure 7a) [69]. In addition, the accumulation of transition-type nucleotide changes is also favored by host cellular mechanisms. These mechanisms principally rely on direct nucleic acid editing through innate immune system-activated antiviral proteins belonging to adenosine deaminase acting on RNA (ADAR) and apolipoprotein-B mRNA-editing catalytic polypeptide-like (APOBEC) families (Figure 7b,c) [25]. Beyond the antiviral activity of some members of these enzyme families, they have also been implicated in the evolution of several viruses, including HIV-1, HBV and SARS-CoV-2, through the generation of viral genetic diversity [70,71]. Antiviral selection may subsequently act upon this genetic diversity, as observed in HIV-1 variants encoding Vif proteins with suboptimal anti-APOBEC3G activity, which give rise to proviruses with LMV drug resistance-associated mutations before any drug exposure [72].

Consequently, it can be deduced that resistance pathways involving transition-type substitutions are associated with a lower genetic barrier than those requiring transversions. Therefore, assessing the genetic barrier should consider not only the number of required substitutions but, more critically, their specific type.

Even when RAS cause a significant reduction in viral fitness, sustained replication under ongoing drug selective pressure can lead to the acquisition of compensatory—or secondary—mutations. These compensatory mutations allow for more efficient viral replication in the presence of the drug, thereby further reducing drug susceptibility [74]. A notable example is observed during the treatment of HCV with sofosbuvir, a highly potent inhibitor of the RNA-dependent RNA polymerase NS5B. The S282T RAS is known to confer high-level resistance to sofosbuvir but incurs a substantial fitness cost [75]. Consequently, with the introduction of new DAAs around 2014, the prevailing clinical opinion was that S282T emergence would be highly improbable due to this fitness cost. However, this assumption was challenged by later studies [76], which demonstrated that mutations such as L159F may alter the position of the amino acid S282 side chain. This mutation can act as a secondary mutation which leads to reduced inhibition by sofosbuvir, thereby potentially enabling the persistence and viability of variants harboring the S282T RAS within the HCV quasispecies.

#### Lessons from Viral Quasispecies: Towards Successful Antiviral Strategies

Decades ago, following the discovery of HIV and the development of the first antiretroviral drugs, the initial treatment paradigm advocated for monotherapy, with sequential switching upon the emergence of resistance. However, clinical experience soon revealed that this approach fostered the stepwise selection of multidrug-resistant variants, rapidly exhausting therapeutic options.

Concurrently, Professor Esteban Domingo, a pioneering researcher in viral variability, in collaboration with Professor John J. Holland, proposed a paradigm shift. In a seminal 1989 review [77], Professor Domingo argued that the most effective strategy to combat highly variable RNA viruses was *combination therapy*. This forward-looking concept anticipated what would later become the standard of care for both HIV and HCV treatment [78,79], embodying the principle of “hit early, hit hard”. This strategy aims to suppress viral replication at early stages, thereby reducing the opportunity for viral populations to increase their fitness and become less sensitive to host immune response or antiviral therapies [80].

Subsequent advancements in antiviral therapy have unequivocally validated this strategy. In HCV, early monotherapy regimens with first-generation DAAs led to the rapid selection of RAS, often within a few days of treatment [81,82]. These observations provided compelling evidence that resistant variants pre-existed within the viral quasispecies before therapy initiation, and that antiviral pressure merely favored their swift expansion. Today, this conceptual framework underpins global public health efforts aimed at eliminating HCV as a major threat, with the World Health Organization targeting elimination chronic viral hepatitis C—and also chronic hepatitis B—as public health threat by 2030 [83]. For instance, Spain represents a leading example, having successfully treated and cured more than 170,000 chronically infected individuals through widespread implementation of highly effective combination therapies [84]. It should also be emphasized that both HBV and HCV are major etiological factors in the development of hepatocellular carcinoma (HCC) [85]. Therefore, an appropriate approach for antiviral therapy represents a true contribution to limit HCC global burden.

**Figure 8:**
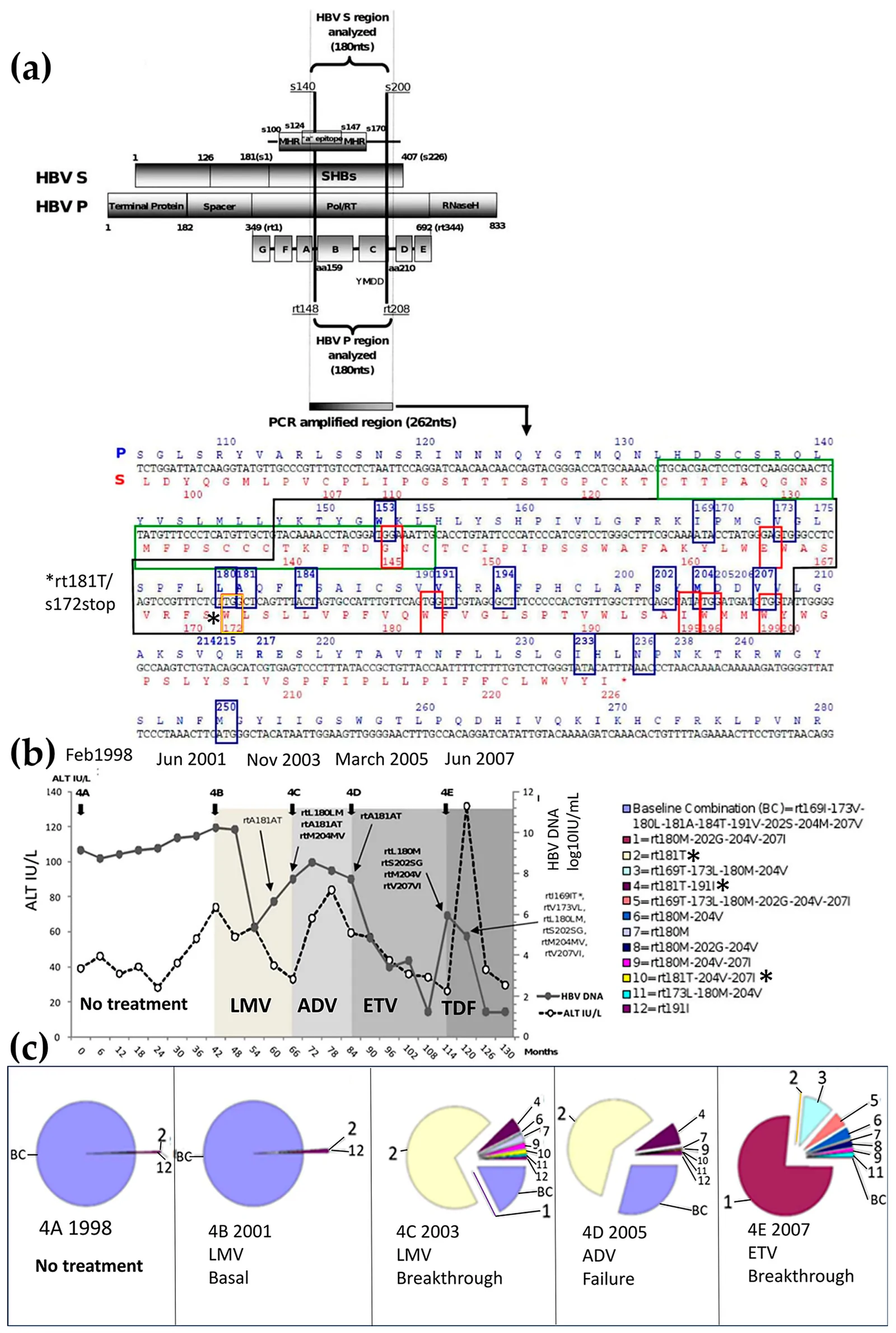
Dynamics of a sequentially treated patient—example from patient 4 in the publication [53]. (**a**) Schematic representation of the overlapping nature of the polymerase (P) and surface (S) open reading frames (ORFs). The fragment of the HBV genome sequence amplified by PCR and analyzed by Ultra-Deep Pyrosequencing (UDPS) is framed in black within the nucleotide sequence. Its translation into amino acids in the P ORF is depicted in blue above the nucleotide sequence, whereas translation in S ORF is depicted in red below. The main codons associated with nucleos(t)ide resistance are framed in blue, while overlapping codons in the S ORF that may give rise to immune escape or premature stop codons are framed in red. Codons affected by the appearance of the rtA181T variant and the overlapping sW172*—stop—are highlighted in yellow. This fragment also encompasses a portion of the C-terminus of the immunodominant epitope “*a*” determinant, framed in green. (**b**) Evolution of alanine amino transferase (ALT) levels and HBV viral load across the five samples analyzed—4A, 4B, 4C, 4D and 4E. For each sample, variants previously detected by reverse hybridization—INNO-LiPA HBV DR v2 assay—and/or direct sequencing, prior to UDPS analysis, are shown. (**c**) Temporal distribution—frequency—of the 12 variants with most variable proportions over the five samples analyzed. Modified from Rodriguez-Frías F, et al [53].

An illustrative example of antiviral escape resulting from the selection of pre-existing variants within an HBV quasispecies comes from a previous longitudinal study [53], in which sequential antiviral treatments revealed the dynamic evolution of an HBV quasispecies. The study demonstrated that resistant variants, which were already present within the quasispecies prior to antiviral therapy—i.e., basal variants—were selected under treatment pressure (Figure 8). In this context, treatment for HBV infection involved either nucleoside analogue polymerase inhibitors, such as LMV or entecavir (ETV), and nucleotide analogue polymerase inhibitors, such as adefovir (ADF) or tenofovir (TDF). As discussed in Section 3.2., the catalytic domain C of the HBV reverse transcriptase contains its putative active site (YMDD), spanning codons 203 to 206. Resistance to LMV is most commonly associated with rtM204V substitution, which alters the YMDD motif to YVDD. In the case examined in this study, the rtM204V mutation likely pre-existed as a rare variant within the HBV quasispecies long before the introduction of NUCs. Consistent with the principles described in Section 3.2, resistant variants may persist at very low frequencies in treatment-naïve individuals despite their reduced replication efficiency, and they often become detectable only through highly sensitive sequencing approaches. In this sense, the frequency of HBV RAS has been analyzed using NGS in several pioneering studies, as summarized in the table reported by Rodriguez-Frías et al. [86].

Figure 8 presents an NGS analysis of HBV quasispecies dynamics in five samples—4A-4E—obtained from the patient examined in this study, using Ultra-Deep Pyrosequencing (UDPS) technology. This patient underwent sequential antiviral treatments [53]. Among the variants identified, the 12 haplotypes—i.e., distinct variants—showing the greatest variability in relative abundance across the five samples analyzed are listed in Figure 8b, alongside variants detected using lower-throughput techniques—as indicated in the graph. The evolutionary dynamics of these haplotypes throughout sequential antiviral therapy, reflecting the selection and emergence of such variants over time, are depicted as sector diagrams, where each sector illustrates the frequency of a specific haplotype (Figure 8c). The changing proportions of these 12 haplotypes provide clear evidence for the selection of pre-existing variants. For instance, haplotype 2, which contained the RAS rtA181T [56], was present as a minor variant in pretreatment samples 4A and 4B and became strongly selected in sample 4C—corresponding to LMV treatment failure. Similarly, haplotype 1, which harbored a combination of RAS including rtL180M, rtS202G and rtM204V [56], was initially present at very low frequency in sample 4C and was undetectable in sample 4D but became strongly selected after ETV treatment failure—sample 4E. These observations clearly indicate that antiviral treatments acted as powerful evolutionary factors, driving selection within the viral quasispecies in a manner that effectively maintains active infection by promoting resistant variants.

The NGS analysis revealed that amino acid substitutions known to be associated with resistance to various NUCs—specifically rtA181T, rtV191I, rtA194T, and rtM204I—were already present at low percentages—even <1%—within the baseline HBV quasispecies, i.e., prior to treatment initiation. Notably, the haplotype 2 in the figure showed the rtA181T RAS and was already detected in pretreatment samples 4A and 4B. This RAS confers *cross-resistance*—defined as decreased susceptibility to more than one antiviral drug conferred by the same RAS or combination of RAS [59]—to LMV and ADF [56]. The selection of a haplotype harboring the rtA181T RAS during LMV treatment can therefore explain the lack of response to subsequent treatment with ADF (Figure 8b), despite the virus having never been exposed to this drug before. Moreover, the rtA181T substitution, resulting from a G-to-A transition, is particularly interesting because it generates a premature stop codon—denoted as an asterisk [*], sW172*, in the overlapping Surface (S) open reading frame (ORF) (Figure 8a, highlighted in yellow). As a consequence, variants carrying this substitution—which became predominant in samples 4C and 4D (Figure 8c)—encode S proteins lacking the last 55 amino acids of their C-terminal region. This truncation renders this variant defective in viral particle secretion and has a negative effect on viral particle expression when co-expressed with WT variants in vitro [87]. Consequently, the formation of complete viral particles by these defective genomes requires the contribution of envelope proteins from other variants within the quasispecies that do not harbor this substitution. This cooperative mechanism is known as *transcomplementation*, a concept that will be further elaborated with additional detailed examples in subsequent sections. Another significant finding was the detection in sample 4C—LMV breakthrough—of a variant combining the RAS rtL180M-rtS202G-rtM204V within the same sequence—haplotype 1 (Figure 8). This combination is known to confer resistance to ETV [56], and accordingly, this haplotype became strongly selected in sample 4E—ETV breakthrough—under ongoing treatment pressure.

### 3.3. New Lessons from Old Data: A Powerful Scientific Approach

In this section, previously published data are reanalyzed to acquire new information. This reanalysis explores the intricate dynamics of HBV quasispecies in the context of antiviral treatment, highlighting the critical role of viral population dynamics in shaping therapeutic outcomes. It also aims to further clarify the quasispecies concept and to illustrate its relevance through compelling clinical examples. In fact, reexamining existing data using novel technologies or updated analytical frameworks can yield profound insights, much like the reanalysis of ancient archeological findings with modern techniques. A notable example is the 2016 analysis of Tutankhamun’s dagger using X-ray fluorescence spectrometry, which revealed its meteoritic origin [88]. Applying a similar principle, the present work revisits HBV viral population data from the influential study by Shirvani-Dastgerdy et al. [89], obtained by means of NGS, to elucidate the relevance of the quasispecies concept in understanding viral infections and their relationship with antiviral treatments. This reanalysis is detailed in the following sections, which illustrate both the selection-driven emergence of antiviral resistance as well as the phenomenon of transcomplementation within the HBV quasispecies dynamics.

#### 3.3.1. Case Studies: HBV Quasispecies Dynamics Under Antiviral Pressure

**Figure 9:**
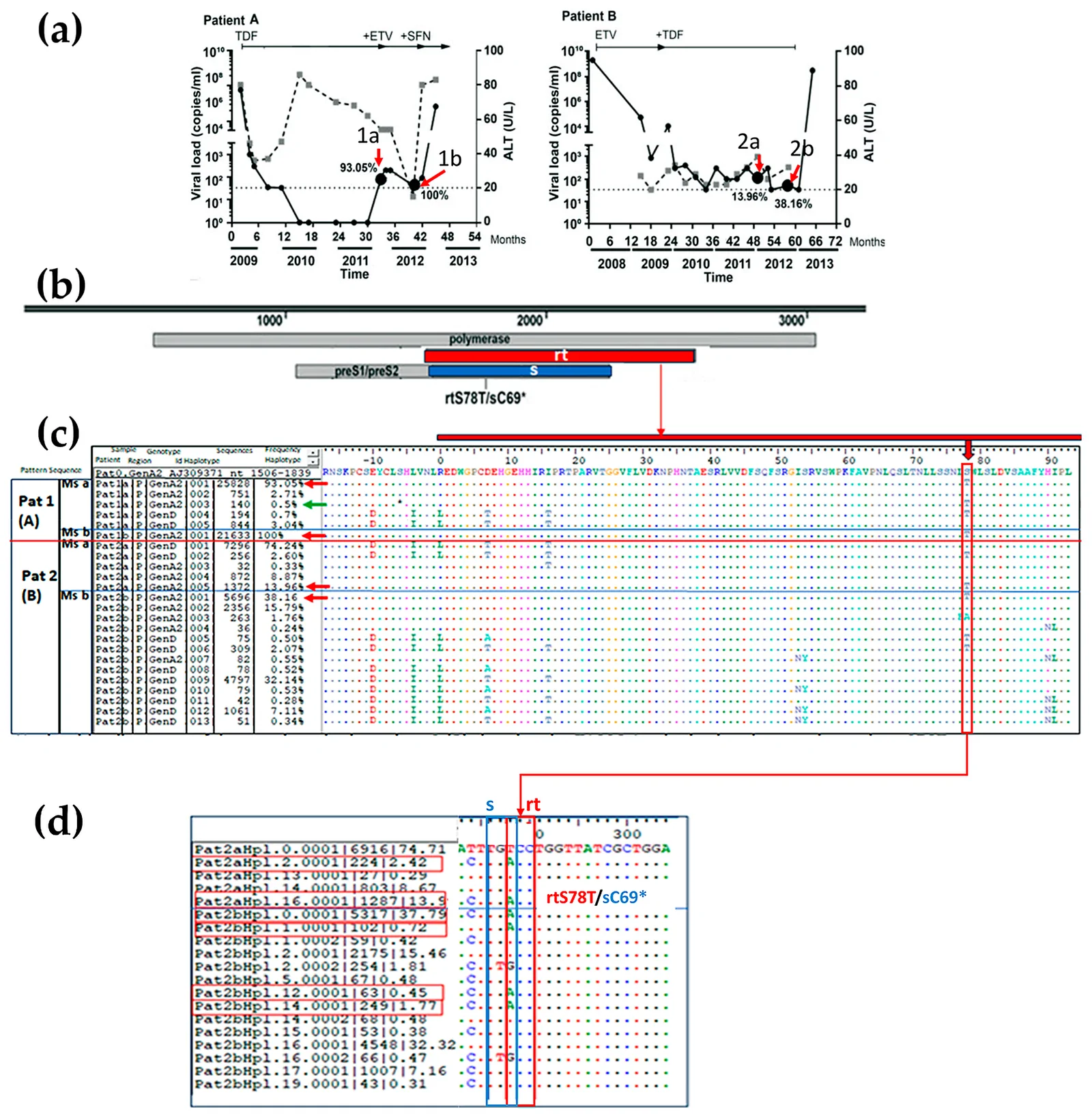
Key findings from the study by Shirvani-Dastgerdy et al. [89]. (**a**) Clinical course of the two patients. Arrows indicate sampling points (1a) and (1b), (2a) and (2b) for each patient, together with the proportions of the rtS78T variant detected at each point. ETV; entecavir, TDF; tenofovir, SFN; sorafenib. (**b**) Schematic representation of the HBV genome, highlighting the reverse transcriptase region (rt)—shown in red—and its overlap with the surface proteins (S) open reading frame—shown in blue. (**c**) Amino acid alignment of the reverse transcriptase region analyzed by means of NGS for the four samples studied from both patients, Patient A—denoted as Pat 1 in the alignment—and Patient B—denoted as Pat 2. Red arrows within this alignment indicate the master—majority—sequences for each patient and sample—Ms a and Ms b. The green arrow highlights the haplotype containing an additional stop codon in the polymerase region—* at position -6 relative to reverse transcriptase numbering, named as Pat1a.P.GenA2.003. (**d**) Nucleotide alignment for Patient B (Pat 2). It specifically highlights the overlapping open reading frames of the surface—framed with a blue box—and reverse transcriptase—framed with a red box—at the nucleotide positions involved in the rtS78T/sC69* substitution. The T-to-A substitution responsible for the amino acid changes is shown in some haplotypes, which names are framed in red. Modified from Journal of Hepatology, 67, Shirvani-Dastgerdi E. et al., Selection of the highly replicative and partially multidrug-resistant rtS78T HBV polymerase mutation during TDF-ETV combination therapy, 246–254, 2017, with permission from Elsevier.

This reanalysis focuses on two illustrative cases of chronic HBV infection included in the previously mentioned study by Shirvani-Dastgerdy et al. (Figure 9), showcasing how antiviral treatments act as powerful evolutionary forces shaping viral quasispecies dynamics. This study describes the selection of HBV variants carrying the rtS78T/sC69* mutation during long-term antiviral therapy in two patients with chronic HBV infection. While the emergence of the rtS78T/sC69* mutation has been occasionally reported in the literature [90,91,92], the present analysis places special emphasis on its particular clinical and virological significance.

- **Patient A (Pat 1):** A 53-year-old HBeAg-negative Caucasian male with HBV-associated HCC initially responded well to TDF therapy post-HCC resection, maintaining undetectable HBV DNA for 21 months. However, approximately 2.5 years later—March 2012—viral rebound occurred in the absence of HCC recurrence. Subsequent combination therapy with ETV and TDF failed to suppress viral replication, and HCC recurred in June 2012, three months after initiating dual therapy. Despite treatment with sorafenib (SFN) was introduced thereafter, the patient’s condition proved fatal.
  This case underscores that “undetectability” is often technique-dependent and that complete viral eradication is a highly challenging, if not elusive, goal. Even with potent antiviral therapies, minority viral populations may persist below the detection threshold and later re-emerge. This phenomenon is conceptually akin to the survival of resilient minority populations of early humans during ancient near-extinction events.
  For instance, the human population is estimated to have been reduced to approximately 1000 individuals about 800,000–900,000 years ago, representing a survival rate of merely 1% from an estimated original population of 100,000 [93]. Despite this dramatic bottleneck, our species not only persisted but subsequently underwent significant expansion over the past 100,000 years, culminating in a global population exceeding 8 billion people. This remarkable demographic recovery underscores how a small fraction of the original population, presumably “resistant” to the prevailing selective pressures, enabled our species’ long-term survival. Metaphorically, this has allowed humans to “infect” the planet on a global scale.
- **Patient B (Pat 2):** A 41-year-old HBeAg-positive Asian female with a high baseline HBV viral load—2 × 10^9^ copies/mL—showed a slow decrease in viral load after ETV initiation. However, an approximately 1 log increase in viral load after 18 months prompted addition of TDF. Despite subsequent dual ETV/TDF therapy, HBV DNA levels were never fully suppressed.

#### 3.3.2. NGS Analysis Reveals Selection of the rtS78T/sC69* Variant

NGS analysis of the viral populations from both patients provides crucial insights into HBV quasispecies evolution. In both patients, a T-to-A nucleotide substitution was detected within the reverse transcriptase region, affecting the sequence encoding the catalytic domain A—between positions rt75 and rt91 [94], leading to the rtS78T mutation. The same nucleotide substitution simultaneously creates a premature stop codon, sC69*, in the overlapping S ORF, due to the complete overlap between the polymerase and viral envelope genes—P and S ORFs—in this genomic region. This sC69* mutation effectively abrogates the synthesis of almost the entire small HBV surface protein and causes inhibition of virion replication and secretion, a defect that can be rescued when sC69* S proteins are co-expressed with WT [95]. In Patient A—Pat 1 in Figure 9c—the rtS78T/sC69* mutation likely emerged during TDF monotherapy, was further selected during TDF/ETV combination therapy, and was tragically associated with aggressive and ultimately fatal HCC recurrence. In Patient B—Pat 2 in Figure 9c,d—this mutation arose during ETV/TDF combination therapy, again showing expansion under drug pressure.

Crucially, the proportions of this rtS78T/sC69* mutation increased significantly during ETV/TDF treatment in both patients—from 93% to 100% in Patient A, and from 14% to 38% in Patient B (Figure 9a). This clear positive selection indicates that variants with this mutation possess reduced sensitivity to these potent antivirals, which are first-line treatments for chronic HBV infection. Consistently, functional studies by Shirvani-Dastgerdi et al. showed that the rtS78T/sC69* change enhances HBV replication and exhibits partial resistance to both ETV and TDF [89].

The selection of HBV isolates with truncated S proteins, similar to the sC69* described here, has been previously reported during NUC treatment. For example, as already illustrated in Figure 8, strong selection of a variant harboring the rtA181T RAS—linked to the overlapping sW172* (stop) in the S ORF—has been described as cause of LMV and ADF treatment failure [53] (Figure 8c). Moreover, these truncated surface variants have often been linked to oncogenic potential [87,96,97]. Thus, it is plausible that these truncated S variants contributed to the fatal HCC in Patient A, given the high proportion of the sC69* variant—100% after recurrence—detected in this patient (Figure 9a). However, our reanalysis suggests that the emergence of the rtS78T/sC69* mutation requires a highly specific genotypic context—as explained in the following section—and the nucleotide change responsible for this change—a T-to-A transversion—is considerably less probable than a transition—as explained in Section 3.2. Escape from Drug Therapy. Collectively, these factors suggest a possible explanation of why this specific variant is rarely selected, which is fortunate given its dramatic clinical consequences. Although the possible basal presence of this variant was not analyzed in this specific study [89], numerous prior studies suggest it is highly probable that resistant variants are present at very low frequencies in baseline samples from treatment-naïve patients. Such variants can be detected when highly sensitive NGS approaches are applied. In this regard, Rodriguez-Frias F et al. [86] summarized several pioneering studies showing that minor resistant variants were collectively detected in 30 of 53 (56.6%) patients across the included studies. Taken together, these findings indicate that antiviral-resistant variants are commonly present within HBV quasispecies prior to treatment initiation.

#### 3.3.3. Genotypic Context Influences Variant Selection

Reanalysis of the published NGS data, summarized in Figure 9, revealed the coexistence of two HBV genotypes—A2 and D—within the same patient’s quasispecies in 3 out of 4 samples analyzed (Figure 9c). More accurately, genotype composition should be considered a quantitative feature of the quasispecies, requiring assessment of the relative proportions of different genotypes within the quasispecies.

Importantly, Figure 9c shows that the rtS78T mutation was preferentially selected only when associated with genotype A2 haplotypes, but not with genotype D haplotypes. For instance, in Patient B, the rtS78T variant in a genotype A2 context—Pat2a.P.GenA2.005—increased from 13.96% to 38.16%, whereas the same mutation in a genotype D context—Pat2a.P.GenD.002—showed no significant change. These observations suggest that the reduced susceptibility to ETV and TDF conferred by the rtS78T change might require specific additional changes present in the Genotype A2 genetic background, which potentially explains the fortunate rarity of this clinically consequential variant. Such genotype-dependent effects are consistent with epistatic interactions—where the phenotypic impact of a given substitution is shaped by other mutations or the surrounding genetic background—phenomena that are widespread among human and non-human viral pathogens [98].

#### 3.3.4. The Phenomenon of Transcomplementation

As explained above (Section 3.3.2. NGS Analysis Reveals Selection of the rtS78T/sC69* Variant), these sC69*-carrying haplotypes were able to persist and be positively selected despite causing inhibition of virion replication and secretion, unless functionally rescued by variants expressing WT S proteins [95]. Based on our reinterpretation of the published NGS data, the seemingly paradoxical existence of variants carrying sC69* is made possible by their “cooperative” interactions with WT variants. This phenomenon is known as *transcomplementation*: deficient genomes benefit from proteins produced by competent (non-deficient) genomes (helper viruses) within the same quasispecies, allowing them to replicate and be maintained within that virus population [99,100]. HBV infection is characterized by a marked overproduction of S proteins, far exceeding the amounts required for complete viral particles assembly. This gives rise to subviral particles composed solely of S proteins—which constitute the HBsAg—that are found at a 10,000 to 100,000-fold excess over the viral particles in the bloodstream of HBV-infected subjects [101,102]. Consequently, even a small minority of WT HBsAg-producing viruses can supply envelope proteins to defective variants, such as HBV genomes carrying the sC69* stop codon, thereby enabling their assembly, release, and subsequent detection in the patient’s bloodstream. This mechanism can explain why even a very minor population capable of producing WT HBsAg—e.g., in Patient A, representing 6% in the first sample and <0.25% in the second—may still provide sufficient envelope proteins to generate complete virions harboring defective genomes.

Furthermore, Patient A’s quasispecies contained a very low-frequency haplotype (0.5%) in the first sample (1a), designated Pat1a.P.GenA2.003—indicated by a green arrow in Figure 9c, which was not selected in the second sample despite harboring the rtS78T substitution. This lack of selection is most likely attributable to the presence of an additional premature stop codon in the P ORF—located at position −6 relative to reverse transcriptase numbering—which would abrogate the synthesis of the full HBV polymerase polyprotein thereby eliminating reverse transcriptase and RNAse H domains. Consequently, without a functional reverse transcriptase, this variant would be unable to replicate effectively and, crucially, would not be capable of developing antiviral resistance, thus preventing its positive selection under treatment pressure. Therefore, although this low-frequency haplotype corresponded to a highly deficient genome, lacking both functional P and S proteins, its detection within the circulating HBV quasispecies from Patient A confirms that it was replicated, enveloped, and released as a complete virion.

Based on our reinterpretation, transcomplementation provides the most plausible explanation for this apparent “mystery”. This mechanism allows an otherwise replication-incompetent genome to be replicated by a functional polymerase supplied by another member of the quasispecies and enveloped by S proteins also provided by other quasispecies members. Although transcomplementation does not imply intentionality or selected cooperation to benefit other genomes, this dynamic can be viewed as a form of “unconscious solidarity” within the quasispecies—where “the community will help you to exist if you cannot achieve it with your own resources”—which allows these “deficient” particles, that might seem “condemned to not exist”, to persist as part of the circulating viral population.

This “unconscious solidarity” is akin to the benefits of social organization in human evolution. The evolutionary success of *Homo sapiens* is arguably rooted in the formation of large communities and their subsequent organization into cities. This concept resonates with Plato’s assertion in *The Republic*: “The city takes its origin from the impotence of each one of us to be self-sufficient [103].” Similarly, viruses do not exist as isolated entities; rather, multiple viral genomes can infect the same cells, hosts, or populations, where they can engage in a wide range of social interactions [104]. The members of a quasispecies coexist in the cellular ecosystem and the viral products they generate may become functionally available to any quasispecies member requiring them. Thanks to this, the “survival” of individual viral genomes is not exclusively dependent on their own functionality, and even defective haplotypes such as Pat1a.P.GenA2.003 (Figure 9c) can be replicated. Furthermore, larger communities inherently foster greater opportunities for transcomplementation between their members, as they are more likely to contain individuals capable of performing diverse and specialized tasks, from which other members of the same community may benefit. Indeed, it has been proposed that the larger community sizes prevalent in *Homo sapiens* populations, compared to those inferred for Neanderthals, may have contributed to the long-term persistence of our species, whereas the Neanderthal populations became extinct [105]. In this regard, the archeological record suggests that severely disabled individuals survived to advanced ages in early human societies, thanks to prolonged care and communal support from their groups [106,107,108].

This concept is illustrated by archaeological findings from ancient human communities. The Atapuerca archaeological sites, in Spain, which have yielded numerous hominin remains spanning more than one million years of human evolution, provide fossil evidence suggesting that individuals with severe physical disabilities survived to ages that would likely have been impossible without sustained community support. A prime example is “Miguelón”—Cranium 5—whose skull exhibits a deformity resulting from a severe and prolonged oral pathology that would have significantly impeded independent feeding [107]. Yet, his survival strongly suggests that his nutritional needs were adequately met through the support of his community. Another case is the 530,000-year-old Cranium 14, which belonged to “Benjamina”, a girl afflicted with craniosynostosis—an incapacitating congenital condition. Nevertheless, she survived to approximately 10–12 years of age [108], likewise indicating prolonged community care.

A similar pattern has been inferred from the Shanidar 1 skeleton—dated to 45,000–35,000 years ago—discovered in Shanidar Cave, Iraq [106]. This Neanderthal individual was severely disabled, likely suffering from blindness, a withered arm, lameness, and possibly deafness. Nonetheless, this individual survived to an advanced age for that period, estimated to be between 35 and 45 years. As with the Atapuerca examples, such survival strongly suggests prolonged care and support from the surrounding community.

In essence, these findings suggest that much like within a viral quasispecies—where defective genomes persist by relying on functional components produced by other variants—survival in early human communities was not solely determined by individual fitness, but also by cooperative frameworks. This form of functional complementation between individuals illustrates the fundamental principle underlying viral transcomplementation, whereby defective genomes may persist because essential functions are supplied by other members of the quasispecies.

#### 3.3.5. Conclusions: Quasispecies Dynamics and Clinical Relevance in Chronic HBV Infection

Our reinterpretation of the HBV viral population data obtained by means of NGS in the study by Shirvani-Dastgerdy et al. [89] highlights that HBV quasispecies are dynamic and complex populations with remarkable adaptive abilities, often utilizing mechanisms such as transcomplementation. The selection of drug-resistant variants is not solely determined by specific mutations but is also strongly influenced by the broader genetic context and by the collective behavior within the viral “community”. Understanding these intricate dynamics is vital for the development of improved antiviral strategies and for interpreting patients’ clinical outcomes. This is exemplified by the severe HCC observed in Patient A, in whom a high proportion of a variant harboring the rtS78T/sC69* mutation was selected (Figure 9c). Importantly, viral load “undetectability” does not imply eradication; the inherent genetic diversity and functional interactions within the quasispecies allow even defective, yet clinically relevant, variants to persist and contribute to its continued evolution.

In essence, despite focusing on a rare variant, our reinterpretation of this study effectively highlights the unique characteristics and critical importance of viral quasispecies. It emphasizes the need for in-depth analyses integrating intrahost genetic diversity with the evolutionary dynamics of viral quasispecies, moving beyond the examination of isolated nucleotide changes. Understanding the global context of the quasispecies—including each haplotype and its genotype—is essential to interpret rare but highly significant phenomena such as treatment failure.

The use of high-sensitivity sequencing methods, such as NGS, to characterize viral quasispecies through comprehensive analyses of intrahost genetic diversity may enable early prediction of the emergence of problematic variants and help optimize treatment strategies. For instance, in cases like Patient A, where truncated viral envelope proteins may be linked to HCC progression, such in-depth analyses could also support prognosis and guide patient management. Ultimately, reexamining previously published data through fresh analytical perspectives—much like re-reading a familiar book—can reveal novel and compelling insights.

### 3.4. Tous pour Un, et Un pour Tous!

Paraphrasing the celebrated motto of *The Three Musketeers*, the dynamics of viral quasispecies can be effectively summarized by such a sound concept “all for one, and one for all”, as illustrated by the compelling example discussed above.

The groundbreaking work by Raúl Andino’s team [100] elegantly demonstrated that the entire viral population—the quasispecies—rather than individual variants, constitutes the true target of selection. This population-level genetic diversity is critical for viral pathogenesis. To illustrate this principle, Andino’s group engineered a poliovirus with a high-fidelity RNA polymerase—harboring the G64S substitution. While this modified virus replicated effectively in vitro, at levels comparable to the WT virus, it generated significantly less genetic diversity within its population. When this less diverse poliovirus was used in vivo to infect animals, the outcome was striking: it caused a less severe disease and, crucially, lost its neurotropism, becoming far less able to infect the central nervous system. In a pivotal subsequent “reverse experiment”, which solidified the causal link between diversity and virulence, Andino’s team artificially boosted the quasispecies diversity of this attenuated, high-fidelity virus through chemical mutagenesis before infection. This forced re-diversification restored both full pathogenic potential and neurotropism of the virus. Further analysis of viruses isolated from infected animal brains revealed complementation among different quasispecies members. These findings showed that even defective or less-fit variants within a diverse viral population can cooperate to achieve a successful infection, underscoring that selection acts on the collective rather than on individual genomes and revealing a genuine form of “social solidarity” within the viral population.

In conclusion, the Andino et al. study demonstrated that the genetic diversity of an RNA viral quasispecies is far more than a mere consequence of high mutation rates. Rather, it constitutes an essential determinant of viral pathogenesis, directly impacting the virus’s capacity to adapt, survive adverse conditions, and successfully infect target tissues. The findings strongly support the view that the viral population as a whole functions as a single unit of selection, with cooperative interactions among its diverse members contributing to the overall viral fitness and pathogenesis. These interactions can take multiple forms, such as “symbiotic” relationships, horizontal genetic transfer, recombination and mutation. Consequently, viral populations are not hierarchically organized like a branching tree but instead resemble “rhizomatic extensions of a plant” [109], a structure defined by multiple, non-hierarchical connections that allow viral variants to interact, complement each other, and collectively shape evolutionary outcomes.

Nevertheless, while these principles are broadly applicable, in HBV infection the full biological and clinical implications of the inherent complexity of HBV quasispecies still await full elucidation through advanced technologies and dedicated research efforts.

### 3.5. Is There Any Evidence of a Quasispecies-like Dynamics Beyond Viruses? Some Hints from the Immune System and Cancer

Up to this point, we have discussed the quasispecies nature of RNA viruses and some DNA viruses, as well as the biological and clinical implications of this concept. In a broader biological context, virtually any replicating genomic entity may exhibit population-level dynamics reminiscent of a viral quasispecies—characterized by high diversity, cooperation or competition among variants, and selection acting on pre-existing heterogeneity.

The adaptive immune system offers a clear nonviral example of such quasispecies-like behavior. Adaptive immunity is mediated by lymphocytes—B and T cells—which can recognize and respond to an extraordinarily large diversity of pathogens. This capacity relies on the huge diversity of their cell surface antigen receptors, B cell receptors (BCRs) and T cell receptors (TCRs), respectively. This diversity is generated through recombination mechanisms acting on pre-existing elements—the variable (V), diversity (D), and joining (J) gene segments—that create variations in the antigen-recognition regions of these receptors. The random nature of this recombination mechanism—called V(D)J recombination—gives rise to a vast pre-immune repertoire of BCRs and TCRs [110].

In B cells, rearrangement of V, D and J segments in the immunoglobulin genes occurs during the early bone marrow-dependent stages of cell development, resulting in a vast pre-immune repertoire of unique clones expressing antibodies capable of recognizing more than 5 × 10^13^ distinct antigenic specificities not previously encountered by the host [111]. A similar principle applies to the TCR repertoire. Recombination of V, D and J gene segments encoding the TCRα and β chains yields an estimated potential repertoire of ~10^19^ unique TCRs (upper theoretical bound). However, the effective TCR repertoire in any individual is constrained by T cell population size, which relies on the thymic production of naïve T cells—estimated at 10^7^ to 10^8^ per day in young adults—and peripheral proliferation [112]. Upon antigen recognition, B and T cells bearing BCRs or TCRs with sufficient affinity are selectively expanded and differentiate into specialized subsets to fight against the invading pathogens [113].

Functionally, these highly diverse B and T cell populations behave as nonviral quasispecies, in which selection acts upon pre-existing specific clones once their matching antigen enters the system. This dynamic closely parallels the evolution of viral quasispecies under selective pressures such as antiviral treatments: variants pre-existing in the population—such as drug-resistant polymerases, rather than mutations arising during treatment—are selected and expanded. Together, these observations underscore that quasispecies-like dynamics are intrinsic not only to viruses but also to human physiology.

While the adaptive immune system represents a tightly regulated, physiological example of quasispecies-like dynamics, comparable principles can also emerge in pathological contexts, such as tumor cell populations. These cells replicate under conditions of genomic instability, often associated with defective DNA repair mechanisms [114], leading to a high degree of genetic variation [115,116]. As Nguyen et al. stated in *Nature Communications* “a given cancer type can display tremendous variation from patient to patient, while within a patient, individual neoplastic lesions often grow at different rates and respond differentially to the same therapy. Even within a given tumor, individual cells can display substantial variation at the genetic, epigenetic and phenotypic levels [117].” As in viral quasispecies exposed to antiviral drugs, this pre-existing intrapopulation heterogeneity enables the selective expansion of resistant clones under therapeutic pressure, even when such clones are present at low frequencies in the primary cancer [118].

So far, we have shown that biological systems displaying high replication rates—such as the adaptive immune system and tumor cells discussed above—can exhibit quasispecies-like population dynamics. However, evolutionary dynamics reminiscent of viral quasispecies—namely, selection acting on pre-existing genetic diversity—can be observed at an even broader scale. For instance, adaptation in recent human evolution often relies on selective pressures acting on genetic variation already present within a population prior to the onset of those pressures [119], reinforcing the centrality of heterogeneity as a fundamental evolutionary principle of biological systems.

## 4. Key Points

The profound implications of viral quasispecies for both medical science and human health demand a fundamental re-evaluation of how viral infections are conceptualized, diagnosed, and treated. Six pivotal conclusions emerge from this framework:

- **Pervasive Genetic Diversity:** Far from being uniform entities, viral populations—particularly RNA viruses and certain DNA viruses such as HBV—exist as intricate, dynamic constellations of closely related yet genetically distinct variants, collectively termed quasispecies. This intrinsic heterogeneity stems directly from their high mutation rates coupled with prodigious replication capacities and results in dynamically shifting population structures.
- **Adaptive Reservoirs for Resistance:** A viral quasispecies acts as a repository of genetic variants. This means that mutations capable of conferring resistance to antiviral agents or enabling immune evasion are frequently present within minority populations long before any selective pressure is exerted. Consequently, antiviral treatment or immune responses do not create these mutations de novo; rather, they preferentially select and amplify pre-existing advantageous variants.
- **Explaining Therapeutic Shortcomings:** This population-based view elucidates why monotherapy often proves insufficient. Resistant variants, even in minor proportions, can swiftly proliferate under selective pressure, and ultimately dominate the quasispecies, culminating in viral relapse and treatment failure. These dynamics underscore the imperative for robust, multi-pronged therapeutic strategies.
- **The Imperative of High-Resolution Sequencing:** The heterogeneous nature of viral quasispecies renders analyses based solely on consensus or master sequences insufficient to capture the true structure and genetic diversity of viral populations. Conventional low-sensitivity methodologies such as Sanger sequencing fail to resolve this complexity and may overlook clinically significant minority variants. Conversely, NGS allows comprehensive characterization of intrahost viral populations, enabling early detection of low-frequency resistance-associated mutations and facilitating the study of quasispecies dynamics. This represents an important shift in how viral populations are interpreted in research and clinical settings, with direct implications for therapeutic decision-making, particularly in instances of therapeutic failure or complex comorbidities.
- **Transcomplementation, a Collective Survival Strategy:** Quasispecies dynamics enable intricate interactions among genetic variants, as exemplified by transcomplementation. Viral genomes rendered defective—e.g., by premature stop codons in crucial genes—may still be replicated and propagated through the supply of functional proteins provided by other variants co-infecting the same host cell. This illustrates that individual viral particles are not necessarily isolated functional units; rather, viral fitness emerges as a property of the population as a whole. In this context, the collective population ensures viability and propagation, even for seemingly “impaired” members. This cooperative, community-like strategy enhances overall persistence.
- **A Broader Evolutionary Principle Beyond Viruses:** At a functional level, the quasispecies paradigm transcends the realm of Virology. Its applicability extends to other rapidly evolving replicating systems—including tumor cell populations undergoing clonal evolution or the adaptive immune system, characterized by vast pre-immune receptor diversity—which can display population-level dynamics driven by pre-existing heterogeneity and selection. This broader framework offers valuable insights into disease progression, the emergence of therapeutic resistance, and the potential benefits of multi-targeted intervention strategies across diverse biological contexts, including both viral infections and malignancies.

## Data Availability

No new data were created or analyzed in this study. Data sharing is not applicable to this article.

## Abbreviations

The following abbreviations are used in this manuscript:

HBV	Hepatitis B virus
HCV	Hepatitis C virus
SARS-CoV-2	Severe acute respiratory syndrome coronavirus 2
RFLP	Restriction fragment length polymorphism
NGS	Next-generation sequencing
H	Shannon’s entropy
Mf	Mutation frequency
Q	Nucleotide diversity
TGS	Third-generation sequencing
RAS	Resistance-associated substitutions
DAA	Direct-acting antivirals
HBsAg	Hepatitis B surface antigen
NUC	Nucleos(t)ide analogues
LMV	Lamivudine
HIV	Human immunodeficiency virus
ALT	Alanine amino transferase
WT	Wild type
ADAR1	Adenosine deaminase acting on RNA, type 1
APOBEC	Apolipoprotein B mRNA-editing enzyme, catalytic polypeptide-like
HCC	Hepatocellular carcinoma
ETV	Entecavir
ADF	Adefovir
TDF	Tenofovir
UDPS	Ultra-deep pyrosequencing
P	Polymerase
S	Surface
ORF	Open reading frame
SFN	Sorafenib
BCR	B cell receptor
TCR	T cell receptor
V	Variable gene segments
D	Diversity gene segments
J	Joining gene segments

## References

[1] Domingo E., Perales C. (2019). Viral Quasispecies. PLoS Genet..

[2] Domingo E., Escarmís C., Lázaro E., Manrubia S.C. (2005). Quasispecies Dynamics and RNA Virus Extinction. Virus Res..

[3] Dreyer T., Haluts A., Korman A., Gov N., Fonio E., Feinerman O. (2025). Comparing Cooperative Geometric Puzzle Solving in Ants versus Humans. Proc. Natl. Acad. Sci. USA.

[4] Peck K.M., Lauring A.S. (2018). Complexities of Viral Mutation Rates. J. Virol..

[5] Gorbalenya A.E., Enjuanes L., Ziebuhr J., Snijder E.J. (2006). Nidovirales: Evolving the Largest RNA Virus Genome. Virus Res..

[6] Park S.G., Kim Y., Park E., Ryu H.M., Jung G. (2003). Fidelity of Hepatitis B Virus Polymerase. Eur. J. Biochem..

[7] Norja P., Eis-Hübinger A.M., Söderlund-Venermo M., Hedman K., Simmonds P. (2008). Rapid Sequence Change and Geographical Spread of Human Parvovirus B19: Comparison of B19 Virus Evolution in Acute and Persistent Infections. J. Virol..

[8] Sheldon J., Rodès B., Zoulim F., Bartholomeusz A., Soriano V. (2006). Mutations Affecting the Replication Capacity of the Hepatitis B Virus. J. Viral Hepat..

[9] Rodríguez-Frías F., Jardi R., Schaper M., Gimferrer M., Elefsiniotis I., Tabernero D., Esteban R., Buti M. (2007). Redetection of HBV Lamivudine-Resistant Mutations in a Patient under Entecavir Therapy, Who Had Been Treated Sequentially with Nucleos(t)Ide Analogues. J. Med. Virol..

[10] Darwin C. On the Origin of Species by Means of Natural Selection, or the Preservation of Favoured Races in the Struggle for Life. https://www.gutenberg.org/cache/epub/1228/pg1228-images.html.

[11] Sanjuán R., Domingo-Calap P. (2016). Mechanisms of Viral Mutation. Cell. Mol. Life Sci..

[12] Townsend P. (2022). The Power of Imperfections.

[13] Cornberg M., Sandmann L., Jaroszewicz J., Kennedy P., Lampertico P., Lemoine M., Lens S., Testoni B., Lai-Hung Wong G., Russo F.P. (2025). EASL Clinical Practice Guidelines on the Management of Hepatitis B Virus Infection. J. Hepatol..

[14] Eigen M., Schuster P. (1979). The Hypercycle: A Principle of Natural Self-Organization.

[15] Eigen M. (1971). Selforganization of Matter and the Evolution of Biological Macromolecules. Naturwissenschaften.

[16] Domingo E., Sabo D., Taniguchi T., Weissmann C. (1978). Nucleotide Sequence Heterogeneity of an RNA Phage Population. Cell.

[17] Lynch M. (2010). Rate, Molecular Spectrum, and Consequences of Human Mutation. Proc. Natl. Acad. Sci. USA.

[18] Colson P., Fantini J., Delerce J., Bader W., Levasseur A., Pontarotti P., Devaux C., Raoult D. (2024). “Outlaw” Mutations in Quasispecies of SARS-CoV-2 Inhibit Replication. Emerg. Microbes Infect..

[19] Perales C., Chen Q., Soria M.E., Gregori J., Garcia-Cehic D., Nieto-Aponte L., Castells L., Imaz A., Llorens-Revull M., Domingo E. (2018). Baseline Hepatitis C Virus Resistance-Associated Substitutions Present at Frequencies Lower than 15% May Be Clinically Significant. Infect. Drug Resist..

[20] Gregori J., Rodríguez-Frías F., Quer J. (2023). Viral Quasispecies Diversity and Evolution A Bioinformatics Molecular Approach.

[21] Modestov A., Buzdin A., Suntsova M. (2025). Unveiling RNA Editing by ADAR and APOBEC Protein Gene Families. Front. Biosci. (Landmark Ed.).

[22] Choi K.H. (2012). Viral Polymerases. Adv. Exp. Med. Biol..

[23] Smith H.C. (2017). RNA Binding to APOBEC Deaminases; Not Simply a Substrate for C to U Editing. RNA Biol..

[24] Steele E.J., Lindley R.A. (2017). ADAR Deaminase A-to-I Editing of DNA and RNA Moieties of RNA:DNA Hybrids Has Implications for the Mechanism of Ig Somatic Hypermutation. DNA Repair.

[25] Ratcliff J., Simmonds P. (2023). The Roles of Nucleic Acid Editing in Adaptation of Zoonotic Viruses to Humans. Curr. Opin. Virol..

[26] Chiu C.H., Chao A., Vogel S., Kriegel P., Thorn S. (2023). Quantifying and Estimating Ecological Network Diversity Based on Incomplete Sampling Data. Philos. Trans. R. Soc. B Biol. Sci..

[27] Morris E.K., Caruso T., Buscot F., Fischer M., Hancock C., Maier T.S., Meiners T., Müller C., Obermaier E., Prati D. (2014). Choosing and Using Diversity Indices: Insights for Ecological Applications from the German Biodiversity Exploratories. Ecol. Evol..

[28] Quarleri J.F., Bussy M.V., Mathet V.L., Ruiz V., Iácono R., Lu L., Robertson B.H., Oubiña J.R. (2003). In Vitro Detection of Dissimilar Amounts of Hepatitis C Virus (HCV) Subtype-Specific RNA Genomes in Mixes Prepared from Sera of Persons Infected with a Single HCV Genotype. J. Clin. Microbiol..

[29] Gregori J., Ibañez-Lligoña M., Quer J. (2023). Quantifying In-Host Quasispecies Evolution. Int. J. Mol. Sci..

[30] Gregori J., Perales C., Rodriguez-Frias F., Esteban J.I., Quer J., Domingo E. (2016). Viral Quasispecies Complexity Measures. Virology.

[31] El País—Elecciones Generales. https://resultados.elpais.com/elecciones/2019-28A/generales/congreso/index.html.

[32] Colomer-Castell S., Gregori J., Garcia-Cehic D., Riveiro-Barciela M., Buti M., Rando-Segura A., Vico-Romero J., Campos C., Ibañez-Lligoña M., Adombi C.M. (2023). In-Host HEV Quasispecies Evolution Shows the Limits of Mutagenic Antiviral Treatments. Int. J. Mol. Sci..

[33] Gregori J., Colomer-Castell S., Ibañez-Lligoña M., Garcia-Cehic D., Campos C., Buti M., Riveiro-Barciela M., Andrés C., Piñana M., González-Sánchez A. (2024). In-Host Flat-like Quasispecies: Characterization Methods and Clinical Implications. Microorganisms.

[34] Gregori J., Colomer-Castell S., Campos C., Ibañez-Lligoña M., García-Cehic D., Cortese M.F., Tabernero D., Riveiro-Barciela M., Buti M., Rando-Segura A. (2026). Peaked-to-Flat Transition in Quasispecies Structure Evolution. Virus Evol..

[35] Somerville V., Lutz S., Schmid M., Frei D., Moser A., Irmler S., Frey J.E., Ahrens C.H. (2019). Long-Read Based de Novo Assembly of Low-Complexity Metagenome Samples Results in Finished Genomes and Reveals Insights into Strain Diversity and an Active Phage System. BMC Microbiol..

[36] Li J., Wang M., Yu D., Han Y., Yang Z., Wang L., Zhang X., Liu F. (2017). A Comparative Study on the Characterization of Hepatitis B Virus Quasispecies by Clone-Based Sequencing and Third-Generation Sequencing. Emerg. Microbes Infect..

[37] Trémeaux P., Latour J., Ranger N., Ferrer V., Harter A., Carcenac R., Boyer P., Demmou S., Nicot F., Raymond S. (2023). SARS-CoV-2 Co-Infections and Recombinations Identified by Long-Read Single-Molecule Real-Time Sequencing. Microbiol. Spectr..

[38] Cook R., Brown N., Rihtman B., Michniewski S., Redgwell T., Clokie M., Stekel D.J., Chen Y., Scanlan D.J., Hobman J.L. (2024). The Long and Short of It: Benchmarking Viromics Using Illumina, Nanopore and PacBio Sequencing Technologies. Microb. Genom..

[39] Luo X., Kang X., Schönhuth A. (2022). Strainline: Full-Length de Novo Viral Haplotype Reconstruction from Noisy Long Reads. Genome Biol..

[40] Mani M., Clipman S.J., Bailey J.R., Sterling R.K., Sulkowski M.S., Anderson M., Olivo A., Belluccini G., Cloherty G., Ribeiro R.M. (2026). Single-Cell Hepatitis B Sequencing Reveals Distinct Viral Infection Events Consistent with Superinfection. J. Infect. Dis..

[41] McElroy K., Thomas T., Luciani F. (2014). Deep Sequencing of Evolving Pathogen Populations: Applications, Errors, and Bioinformatic Solutions. Microb. Inform. Exp..

[42] Lu I.-N., Muller C.P., He F.Q. (2020). Applying Next-Generation Sequencing to Unravel the Mutational Landscape in Viral Quasispecies. Virus Res..

[43] Cammack R., Atwood T., Campbell P., Parish H., Smith A., Vella F., Stirling J. (2006). Oxford Dictionary of Biochemistry and Molecular Biology.

[44] Domingo E., Holland J.J. (1997). RNA Virus Mutations and Fitness for Survival. Annu. Rev. Microbiol..

[45] Domingo E., Soria M.E., Gallego I., de Ávila A.I., García-Crespo C., Martínez-González B., Gómez J., Briones C., Gregori J., Quer J. (2020). A New Implication of Quasispecies Dynamics: Broad Virus Diversification in Absence of External Perturbations. Infect. Genet. Evol..

[46] Gallego I., Gregori J., Soria M.E., García-Crespo C., García-Álvarez M., Gómez-González A., Valiergue R., Gómez J., Esteban J.I., Quer J. (2018). Resistance of High Fitness Hepatitis C Virus to Lethal Mutagenesis. Virology.

[47] Gallego I., Soria M.E., García-Crespo C., Chen Q., Martínez-Barragán P., Khalfaoui S., Martínez-González B., Sanchez-Martin I., Palacios-Blanco I., de Ávila A.I. (2020). Broad and Dynamic Diversification of Infectious Hepatitis C Virus in a Cell Culture Environment. J. Virol..

[48] Moreno E., Gallego I., Gregori J., Lucía-Sanz A., Soria M.E., Castro V., Beach N.M., Manrubia S., Quer J., Esteban J.I. (2017). Internal Disequilibria and Phenotypic Diversification during Replication of Hepatitis C Virus in a Noncoevolving Cellular Environment. J. Virol..

[49] Sheldon J., Beach N.M., Moreno E., Gallego I., Piñeiro D., Martínez-Salas E., Gregori J., Quer J., Esteban J.I., Rice C.M. (2014). Increased Replicative Fitness Can Lead to Decreased Drug Sensitivity of Hepatitis C Virus. J. Virol..

[50] Mathet V.L., López J.L., Ruiz V., Sánchez D.O., Carballal G., Campos R.H., Oubiña J.R. (2007). Dynamics of a Hepatitis B Virus e Antigen Minus Population Ascribed to Genotype F during the Course of a Chronic Infection despite the Presence of Anti-HBs Antibodies. Virus Res..

[51] López J.L., Mathet V.L., Oubiña J.R., Campos R.H. (2007). Intrahost Evolution of HBe Antigen-Negative Hepatitis B Virus Genomes Ascribed to the F Genotype: A Longitudinal 3 Year Retrospective Study. J. Gen. Virol..

[52] Tzu S. (2015). The Art of War.

[53] Rodriguez-Frías F., Tabernero D., Quer J., Esteban J.I., Ortega I., Domingo E., Cubero M., Camós S., Ferrer-Costa C., Sánchez A. (2012). Ultra-Deep Pyrosequencing Detects Conserved Genomic Sites and Quantifies Linkage of Drug-Resistant Amino Acid Changes in the Hepatitis B Virus Genome. PLoS ONE.

[54] Rodriguez C., Chevaliez S., Bensadoun P., Pawlotsky J.-M. (2013). Characterization of the Dynamics of Hepatitis B Virus Resistance to Adefovir by Ultra-Deep Pyrosequencing. Hepatology.

[55] Global HIV, Hepatitis and STIs Programmes (HHS), Guidelines Review Committee (2024). Guidelines for the Prevention, Diagnosis, Care and Treatment for People with Chronic Hepatitis B Infection.

[56] Warner N., Locarnini S. (2014). Mechanisms of Hepatitis B Virus Resistance Development. Intervirology.

[57] Yeh C.-T., Chien R.-N., Chu C.-M., Liaw Y.-F. (2000). Clearance of the Original Hepatitis B Virus YMDD-Motif Mutants with Emergence of Distinct Lamivudine-Resistant Mutants during Prolonged Lamivudine Therapy. Hepatology.

[58] Melegari M., Scaglioni P.P., Wands J.R. (1998). Hepatitis B Virus Mutants Associated with 3TC and Famciclovir Administration Are Replication Defective. Hepatology.

[59] Lok A.S., Zoulim F., Locarnini S., Bartholomeusz A., Ghany M.G., Pawlotsky J.-M., Liaw Y.-F., Mizokami M., Kuiken C. (2007). Antiviral Drug-Resistant HBV: Standardization of Nomenclature and Assays and Recommendations for Management. Hepatology.

[60] European Association for the Study of the Liver (2017). EASL 2017 Clinical Practice Guidelines on the Management of Hepatitis B Virus Infection. J. Hepatol..

[61] Vermehren J., Sarrazin C. (2012). The Role of Resistance in HCV Treatment. Best Pract. Res. Clin. Gastroenterol..

[62] Sorbo M.C., Cento V., Di Maio V.C., Howe A.Y.M., Garcia F., Perno C.F., Ceccherini-Silberstein F. (2018). Hepatitis C Virus Drug Resistance Associated Substitutions and Their Clinical Relevance: Update 2018. Drug Resist. Updates.

[63] Howe A.Y.M., Rodrigo C., Cunningham E.B., Douglas M.W., Dietz J., Grebely J., Popping S., Sfalcin J.A., Parczewski M., Sarrazin C. (2022). Characteristics of Hepatitis C Virus Resistance in an International Cohort after a Decade of Direct-Acting Antivirals. JHEP Rep..

[64] Lenz O., Vijgen L., Berke J.M., Cummings M.D., Fevery B., Peeters M., De Smedt G., Moreno C., Picchio G. (2013). Virologic Response and Characterisation of HCV Genotype 2–6 in Patients Receiving TMC435 Monotherapy (Study TMC435-C202). J. Hepatol..

[65] Vicenti I., Rosi A., Saladini F., Meini G., Pippi F., Rossetti B., Sidella L., Di Giambenedetto S., Almi P., De Luca A. (2012). Naturally Occurring Hepatitis C Virus (HCV) NS3/4A Protease Inhibitor Resistance-Related Mutations in HCV Genotype 1-Infected Subjects in Italy. J. Antimicrob. Chemother..

[66] Kuntzen T., Timm J., Berical A., Lennon N., Berlin A.M., Young S.K., Lee B., Heckerman D., Carlson J., Reyor L.L. (2008). Naturally Occurring Dominant Resistance Mutations to Hepatitis C Virus Protease and Polymerase Inhibitors in Treatment-Naïve Patients. Hepatology.

[67] Cubero M., Esteban J.I., Otero T., Sauleda S., Bes M., Esteban R., Guardia J., Quer J. (2008). Naturally Occurring NS3-Protease-Inhibitor Resistant Mutant A156T in the Liver of an Untreated Chronic Hepatitis C Patient. Virology.

[68] Bartels D.J., Zhou Y., Zhang E.Z., Marcial M., Byrn R.A., Pfeiffer T., Tigges A.M., Adiwijaya B.S., Lin C., Kwong A.D. (2008). Natural Prevalence of Hepatitis C Virus Variants with Decreased Sensitivity to NS3·4A Protease Inhibitors in Treatment-Naive Subjects. J. Infect. Dis..

[69] Wakeley J. (1996). The Excess of Transitions among Nucleotide Substitutions: New Methods of Estimating Transition Bias Underscore Its Significance. Trends Ecol. Evol..

[70] Piontkivska H., Wales-McGrath B., Miyamoto M., Wayne M.L. (2021). ADAR Editing in Viruses: An Evolutionary Force to Reckon with. Genome Biol. Evol..

[71] Sadeghpour S., Khodaee S., Rahnama M., Rahimi H., Ebrahimi D. (2021). Human APOBEC3 Variations and Viral Infection. Viruses.

[72] Mulder L.C.F., Harari A., Simon V. (2008). Cytidine Deamination Induced HIV-1 Drug Resistance. Proc. Natl. Acad. Sci. USA.

[73] Li F., Zhang J., Kang J., Tong X., Zhang P., Zhu Y. (2025). Inosine: Biofunctions and the Roles in Human Diseases. Front. Endocrinol..

[74] Irwin K.K., Renzette N., Kowalik T.F., Jensen J.D. (2016). Antiviral Drug Resistance as an Adaptive Process. Virus Evol..

[75] Walker A., Filke S., Lübke N., Obermeier M., Kaiser R., Häussinger D., Timm J., Bock H.H. (2017). Detection of a Genetic Footprint of the Sofosbuvir Resistance-Associated Substitution S282T after HCV Treatment Failure. Virol. J..

[76] Donaldson E.F., Harrington P.R., O’Rear J.J., Naeger L.K. (2015). Clinical Evidence and Bioinformatics Characterization of Potential Hepatitis C Virus Resistance Pathways for Sofosbuvir. Hepatology.

[77] Domingo E. (1989). RNA Virus Evolution and the Control of Viral Disease. Progress in Drug Research.

[78] Pawlotsky J.M. (2014). New Hepatitis C Therapies: The Toolbox, Strategies, and Challenges. Gastroenterology.

[79] Palella F.J., Delaney K.M., Moorman A.C., Loveless M.O., Fuhrer J., Satten G.A., Aschman D.J., Holmberg S.D., HIV Outpatient Study Investigators (1998). Declining Morbidity and Mortality among Patients with Advanced Human Immunodeficiency Virus Infection. N. Engl. J. Med..

[80] Domingo E. (2020). Quasispecies Dynamics in Disease Prevention and Control. Virus as Populations.

[81] Sarrazin C., Zeuzem S. (2010). Resistance to Direct Antiviral Agents in Patients with Hepatitis C Virus Infection. Gastroenterology.

[82] Sarrazin C., Kieffer T.L., Bartels D., Hanzelka B., Müh U., Welker M., Wincheringer D., Zhou Y., Chu H.M., Lin C. (2007). Dynamic Hepatitis C Virus Genotypic and Phenotypic Changes in Patients Treated with the Protease Inhibitor Telaprevir. Gastroenterology.

[83] Global HIV, Hepatitis and STIs Programmes (HHS) (2022). Global Health Sector Strategies on, Respectively, HIV, Viral Hepatitis and Sexually Transmitted Infections for the Period 2022–2030.

[84] Spanish Health Ministry More Than 172,000 Patients Have Been Treated and Cured of Hepatitis C in Spain in a Decade. https://www.lamoncloa.gob.es/serviciosdeprensa/notasprensa/sanidad14/paginas/2025/110425-garcia-pacientes-hepatitis-c.aspx.

[85] Forner A., Reig M., Bruix J. (2018). Hepatocellular Carcinoma. Lancet.

[86] Rodriguez-Frias F., Tabernero D., Esteban R., Buti M. (2014). Study of Hepatitis B Virus Quasispecies by Ultra-Deep Pyrosequencing: Resolving the Nitty-Gritty. Hepatology.

[87] Warner N., Locarnini S.A. (2008). The Antiviral Drug Selected Hepatitis B Virus RtA181T/SW172* Mutant Has a Dominant Negative Secretion Defect and Alters the Typical Profile of Viral Rebound. Hepatology.

[88] Comelli D., D’orazio M., Folco L., El-Halwagy M., Frizzi T., Alberti R., Capogrosso V., Elnaggar A., Hassan H., Nevin A. (2016). The Meteoritic Origin of Tutankhamun’s Iron Dagger Blade. Meteorit. Planet. Sci..

[89] Shirvani-Dastgerdi E., Winer B.Y., Celià-Terrassa T., Kang Y., Tabernero D., Yagmur E., Rodríguez-Frías F., Gregori J., Luedde T., Trautwein C. (2017). Selection of the Highly Replicative and Partially Multidrug Resistant RtS78T HBV Polymerase Mutation during TDF-ETV Combination Therapy. J. Hepatol..

[90] Xiang K.-h., Michailidis E., Ding H., Peng Y.-q., Su M.-z., Li Y., Liu X.e., Dao Thi V.L., Wu X.f., Schneider W.M. (2016). Effects of Amino Acid Substitutions in Hepatitis B Virus Surface Protein on Virion Secretion, Antigenicity, HBsAg and Viral DNA. J. Hepatol..

[91] Betz-Stablein B.D., Töpfer A., Littlejohn M., Yuen L., Colledge D., Sozzi V., Angus P., Thompson A., Revill P., Beerenwinkel N. (2016). Single-Molecule Sequencing Reveals Complex Genome Variation of Hepatitis B Virus during 15 Years of Chronic Infection Following Liver Transplantation. J. Virol..

[92] Huang S.F., Chen Y.T., Lee W.C., Chang I.C., Chiu Y.T., Chang Y., Tu H.C., Yuh C.H., Matsuura I., Shih L.Y. (2014). Identification of Transforming Hepatitis B Virus S Gene Nonsense Mutations Derived from Freely Replicative Viruses in Hepatocellular Carcinoma. PLoS ONE.

[93] Hu W., Hao Z., Du P., Di Vincenzo F., Manzi G., Cui J., Fu Y.-X., Pan Y.-H., Li H. (2023). Genomic Inference of a Severe Human Bottleneck during the Early to Middle Pleistocene Transition. Science.

[94] Stuyver L.J., Locarnini S.A., Lok A., Richman D.D., Carman W.F., Dienstag J.L., Schinazi R.F. (2001). Nomenclature for Antiviral-Resistant Human Hepatitis B Virus Mutations in the Polymerase Region. Hepatology.

[95] Xiang K., Xiao Y., Li Y., He L., Wang L., Zhuang H., Li T. (2019). The Effect of the Hepatitis B Virus Surface Protein Truncated SC69∗ Mutation on Viral Infectivity and the Host Innate Immune Response. Front. Microbiol..

[96] Lee S.-A., Kim K., Kim H., Kim B.-J. (2012). Nucleotide Change of Codon 182 in the Surface Gene of Hepatitis B Virus Genotype C Leading to Truncated Surface Protein Is Associated with Progression of Liver Diseases. J. Hepatol..

[97] Lai M.-W., Huang S.-F., Hsu C.-W., Chang M.-H., Liaw Y.-F., Yeh C.-T. (2009). Identification of Nonsense Mutations in Hepatitis B Virus S Gene in Patients with Hepatocellular Carcinoma Developed after Lamivudine Therapy. Antivir. Ther..

[98] Manivannan S.N., Diaz Arenas C., Grubaugh N.D., Ogbunugafor C.B. (2025). The Importance of Epistasis in the Evolution of Viral Pathogens. Virus Evol..

[99] Shirogane Y., Watanabe S., Yanagi Y. (2019). Cooperation between Different Variants: A Unique Potential for Virus Evolution. Virus Res..

[100] Vignuzzi M., Stone J.K., Arnold J.J., Cameron C.E., Andino R. (2006). Quasispecies Diversity Determines Pathogenesis through Cooperative Interactions in a Viral Population. Nature.

[101] Hu J., Liu K. (2017). Complete and Incomplete Hepatitis B Virus Particles: Formation, Function, and Application. Viruses.

[102] Bruss V. (2007). Hepatitis B Virus Morphogenesis. World J. Gastroenterol..

[103] Plato, Emlyn-Jones C., Preddy W. (2013). Republic, Volume I: Books 1–5.

[104] Leeks A., Bono L.M., Ampolini E.A., Souza L.S., Höfler T., Mattson C.L., Dye A.E., Díaz-Muñoz S.L. (2023). Open Questions in the Social Lives of Viruses. J. Evol. Biol..

[105] Kolodny O., Feldman M.W. (2017). A Parsimonious Neutral Model Suggests Neanderthal Replacement Was Determined by Migration and Random Species Drift. Nat. Commun..

[106] Trinkaus E. (1983). The Shanidar Neandertals.

[107] Pérez P.J., Gracia A., Martínez I., Arsuaga J.L. (1997). Paleopathological Evidence of the Cranial Remains from the Sima de Los Huesos Middle Pleistocene Site (Sierra de Atapuerca, Spain). Description and Preliminary Inferences. J. Hum. Evol..

[108] Gracia A., Arsuaga J.L., Martínez I., Lorenzo C., Carretero J.M., Bermúdez de Castro J.M., Carbonell E. (2009). Craniosynostosis in the Middle Pleistocene Human Cranium 14 from the Sima de Los Huesos, Atapuerca, Spain. Proc. Natl. Acad. Sci. USA.

[109] Landa C.R., Ariza-Mateos A., Briones C., Perales C., Wagner A., Domingo E., Gómez J. (2024). Adapting the Rhizome Concept to an Extended Definition of Viral Quasispecies and the Implications for Molecular Evolution. Sci. Rep..

[110] Market E., Papavasiliou F.N. (2003). V(D)J Recombination and the Evolution of the Adaptive Immune System. PLoS Biol..

[111] Pieper K., Grimbacher B., Eibel H. (2013). B-Cell Biology and Development. J. Allergy Clin. Immunol..

[112] Weng N. (2023). Numbers and Odds: TCR Repertoire Size and Its Age Changes Impacting on T Cell Functions. Semin. Immunol..

[113] Yatim K.M., Lakkis F.G. (2015). A Brief Journey through the Immune System. Clin. J. Am. Soc. Nephrol..

[114] Cahill D.P., Kinzler K.W., Vogelstein B., Lengauer C. (1999). Genetic Instability and Darwinian Selection in Tumours. Trends Cell Biol..

[115] Napoletani D., Signore M., Struppa D.C. (2013). Cancer Quasispecies and Stem-like Adaptive Aneuploidy. F1000Research.

[116] Campbell P.J., Pleasance E.D., Stephens P.J., Dicks E., Rance R., Goodhead I., Follows G.A., Green A.R., Futreal P.A., Stratton M.R. (2008). Subclonal Phylogenetic Structures in Cancer Revealed by Ultra-Deep Sequencing. Proc. Natl. Acad. Sci. USA.

[117] Nguyen A., Yoshida M., Goodarzi H., Tavazoie S.F. (2016). Highly Variable Cancer Subpopulations That Exhibit Enhanced Transcriptome Variability and Metastatic Fitness. Nat. Commun..

[118] Turner N.C., Reis-Filho J.S. (2012). Genetic Heterogeneity and Cancer Drug Resistance. Lancet Oncol..

[119] Schrider D.R., Kern A.D. (2017). Soft Sweeps Are the Dominant Mode of Adaptation in the Human Genome. Mol. Biol. Evol..

